# Gene-Based Genome-Wide Association Study Identified Genes for Agronomic Traits in Maize

**DOI:** 10.3390/biology11111649

**Published:** 2022-11-11

**Authors:** Yunfeng Zhao, Jin Gao, Xiugang Guo, Baofeng Su, Haijie Wang, Runqing Yang, Li Jiang

**Affiliations:** 1 Key Laboratory of Aquatic Genomics, Ministry of Agriculture and Rural Affairs, Beijing Key Laboratory of Fishery Biotechnology, Chinese Academy of Fishery Sciences, Beijing 100141, China; 2 General Education College, Weifang University of Science and Technology, Weifang 262700, China; 3 Hainan Academy of Ocean and Fisheries Sciences, Haikou 571126, China; 4 School of Fisheries, Aquaculture and Aquatic Sciences, Auburn University, Auburn, AL 36849, USA

**Keywords:** GWAS, SNP, gene, association analysis, maize

## Abstract

**Simple Summary:**

Genome-wide association studies (GWAS) have successfully detected many SNPs related to complex quantitative traits. However, SNPs significantly associated with quantitative traits usually have only mild effects. Quantitative traits are usually caused by the combined effects of multiple loci in a gene. Maize is one of the world’s most important foods and feed crops. Earlier silking, kernel oil concentration, and fatty acid composition are all important agronomic traits in maize. To further explore the gene-level variations affecting maize economic traits, we propose an efficient gene-based GWAS method. We applied this method to the economic traits of maize and identified many candidate genes. Many of the same candidate genes were found in the analysis of related maize traits, which proved the reliability of our method. These findings will provide a theoretical basis for maize breeding with the targeted earlier silking and kernel oil concentration traits.

**Abstract:**

A gene integrates the effects of all SNPs in its sequence span, which benefits the genome-wide association study. To explore gene-level variations affecting economic traits in maize, we extended the SNP-based GWAS analysis software Single-RunKing developed by our team to gene-based GWAS, which used the FaST-LMM algorithm to convert the linear mixed model into simple linear model association analysis. An F-test statistic was formulated to test and identify candidate genes. We compared the statistical efficiency of using 80% principal components (EPC), the first principal component (FPC), and all SNP markers (ALLSNP) as independent variables, which predecessors commonly used to integrate SNPs and represent genes. With a Huazhong Agricultural University (HAU) genomic dataset of 2.65M SNPs from 540 maize plants, 34,774 genes were annotated across the whole genome. Genome-wide association studies with 20 agronomic traits were performed using the software developed here. Another maize dataset from the Ames panel (AP) was also analyzed. The EPC method fits the model well and has good statistical efficiency. It not only overcomes the false negative problem when using all SNP markers for analysis (ALLSNP) but also solves the false positive problem of its corresponding simple linear model method EPCLM. Compared with FPC, the EPC method has higher statistical efficiency. A total of 132 quantitative trait genes (QTG) were identified for the 20 traits from HAU maize dataset and one trait of AP maize.

## 1. Introduction

Genome-wide association studies (GWAS) have successfully detected many SNPs related to complex quantitative traits. However, the method of using single SNPs for association studies has its disadvantages. For example, SNPs that have been shown to be significantly associated with complex diseases usually have only mild effects [1]. Common diseases are usually caused by the combined effects of multiple loci in a gene. If only the significant SNPs are considered, genetic mutations that collectively have a significant impact but individually contribute little will be missed. To examine whether a gene is related to a trait or a disease, many multi-marker association analysis methods have been developed, such as haplotype-based methods [2,3], *p*-value combination methods [4,5,6] and principal component analysis (PCA)-based methods [7,8,9,10]. Studies have shown that PCA-based methods are as effective as or more powerful than the haplotype-based or standard joint SNP tests [10]. Haplotype-based analysis is more computationally demanding compared to PCA-based method [10]. In the *p*-value combination method, it is assumed that the *p*-values are independent. However, this assumption does not fit the actual situation for many practical applications, such as genome-wide association scans using dense SNP markers. With the advancement of sequencing technology and the reduction of cost, a considerable amount of SNP markers was detected, which makes the previous problem more prominent. This problem can be solved by doing PCA because principal components (PC) are orthogonal.

The multi-SNPs or gene-based association studies conducted by predecessors generally choose the top PCs, which accounted for 80–85% of the total variation of SNP data, as independent variables for the following regression analyses [8,10,11]. Yang et al. applied the Fisher’s combination test [12] to the PC independent variables within each gene to get a new *p*-value for the gene [11]. All these PCA-based methods are general linear model methods rather than linear mixed model methods, which showed obvious false positives when applied to the maize data in this research. Yano et al. used a linear mixed model in their research and suggested using the first or the first two PCs for gene-based GWAS analysis [13]. Regarding how many PCs should be used as independent variables, predecessors have done a lot of simulation and actual data research, thus here, we just compare the statistical efficiency of using 80% PCs and the first PC as independent variables.

In linear model regression analysis, results can be confounded by cryptic relatedness and population stratification, resulting in false positive rates. The linear mixed model (LMM) corrects these confounding factors by using random polygenic effects that exclude the tested genetic unit, which can help to effectively control the false positive rates and improve the ability to detect quantitative trait nucleotides (QTN). In addition, people have further improved and simplified the algorithm to reduce the computational intensity of LMM [14,15,16,17,18,19,20,21], thereby reducing the computational burden and making LMM more and more widely used in genome-wide association studies (GWAS). These simplified methods work by reducing the LMM or replacing the restricted maximum likelihood (REML) [22] with spectral decomposition. The reduced LMM methods include the GRAMMAR [14], EMMAX [15] or P3D [16], CMLM [16], GRAMMAR-Gamma [17], and BOLT-LMM [18]. Although these methods keep a similar statistical power as traditional LMM, the residual polygenic effects are overestimated, which leads to a decrease in the goodness of fit to the phenotype. Instead of REML, the efficient mixed-model association (EMMA) [19] uses the spectral decomposition of markers and phenotypes to avoid redundant and intensive matrix computation problems during each iteration in the likelihood function calculation. These speed up the calculation of solving the LMM by several orders of magnitude. However, EMMA needs to do the spectral decomposition of each tested SNP, which still consumes much memory and affects speed. A better option, the factored spectrally transformed linear mixed models (FaST-LMM) [20], just needs one-time spectral decomposition to complete the test for all SNPs, thus solving this problem. Finally, based on the spectral decomposition, the second derivatives of the log-likelihood function are evaluated and used in the genome-wide efficient mixed-model association (GEMMA) [21] method to find the global optimal value.

We used the FaST-LMM algorithm to convert the linear mixed model association analysis into a simple linear model association analysis and constructed an F statistic for the tested genes to make the FaST-LMM used for SNPs detection applicable to genes. In order to accelerate the whole genome regression scan, the fastLmPure function of the R/RcppArmadillo package was integrated into our method to evaluate the effects of the tested genes. If only testing the large or highly significant genes obtained by EMMAX, it can help further reduce the whole genome gene association analysis to one or two rounds of whole genome regression scans. Based on these ideas, the Single-RunKing software [23] was developed by our team to perform the rapid whole genome mixed model association study. We have applied this software to the analysis of haplotypes [24]. However, performing gene-based GWAS analysis would be more biologically meaningful, considering that genes are the basic physical and functional units of heredity that control biological traits [25]. In addition, the results of gene-based GWAS are also helpful for further research on pathway-based GWAS. Based on the Single-RunKing software and further considering the independent variables, we adopted using 80% PCs as the independent variable and named it the EPC method. The PC independent variable for each gene was obtained by performing principal component analysis on the SNPs within each gene block separately. We compared EPC’s efficiency with two other linear mixed model methods: One is the traditional method ALLSNP, which represents each gene with its internal SNPs to perform genome-wide gene association analysis. The other one uses the first PC of each gene as the independent variable to perform the linear mixed model regression analysis with the phenotype, which is named the FPC method. The results of the simple linear model method (EPCLM), which corresponds to the linear mixed model method EPC, are also compared. By reanalyzing twenty traits of the Huazhong Agricultural University (HAU) maize dataset [26] and one trait of the Ames panel (AP) maize dataset [27], the EPC method is proved to be more efficient than ALLSNP, FPC, and EPCLM in terms of model fitting, quantitative trait gene (QTG) identification. We proposed a simple and efficient gene-based GWAS method in this study and identified candidate genes for maize economic traits.

## 2. Materials and Methods

### 2.1. Maize Genomic Data Processing

In this study, we analyzed AP and HAU maize datasets to assess the performance of our proposed method. Due to a large number of individuals in the AP dataset, we used it to carry out simulation experiments. The case analyses were conducted using AP and HAU datasets, respectively. The AP maize dataset consists of 2279 inbred lines, with 681,258 SNPs genotyped. The trait we analyzed is days to silking (DTS). The AP maize datasets are free to download from the website (http://www.panzea.org/#!genotypes/cctl (accessed on 6 November 2022)) [27]. The 540 maize inbred lines of the HAU maize datasets were from a global collection [28], including representative temperate and tropical/subtropical inbred lines. More than 2.65M SNPs were obtained for these 540 individuals, 1.25 M of which had a MAF ≥ 5% and were used for further studies. The HAU maize datasets are publicly available on the website of Jianbing Yan (http://www.maizego.org/Resources.html (accessed on 6 November 2022)) [26]. The traits analyzed were kernel oil concentration and fatty acid composition, measured in multiple environments as described in the previous study [29]. Table 1 lists the details of the 20 agronomic traits in the HAU maize dataset.

Because genes are to be used as genetic units for GWAS analysis, SNP markers need to be assigned to each gene first. Both AP maize data reference genome annotation file ZmB73_5a.59_WGS.gff3 and HAU maize data reference genome annotation file ZmB73_5b.60_FGS.gff were downloaded from the website https://www.maizegdb.org/ (accessed on 6 November 2022). Then, SNP markers were assigned to genes. Multiple SNPs within a gene may collectively have a large effect but individually contribute little. The gene-based GWAS method can find genes containing these minor mutations. Because a certain proportion of genes only contain a few SNPs, in order to focus on the integration effects of genes on their internal SNP effects under different methods, the genes with more than 10 SNP markers were further screened for the following analysis, and their corresponding SNP markers form the final genotype matrix.

The 681,258 SNPs of AP maize were annotated into 37,292 genes, while a total of 16,893 genes with at least 10 SNPs were screened for further gene-based GWAS analysis, covering 347,481 SNPs, accounting for 51.01% of initial SNP markers. This way, the number of remaining genes after screening is not too small, and each gene contains sufficient SNPs. We finally analyzed all genes in the actual case analysis and listed the complete results in Table S3. The 1.25 M SNPs of HAU maize data were correspondingly annotated into 34,774 genes, while a total of 24,594 genes with at least 10 SNPs were screened for further gene-based GWAS analysis, covering 932,712 SNPs, accounting for 74.6% of the initial SNP markers.

### 2.2. FaST-LMM for Genes

The general LMM of GWAS can be expressed in matrix notation as:

y=1μ+Xβ+Za+ε,

where ***y*** is the objective trait of *n* individuals, μ is the population mean, ***β*** is the additive genetic effect of the tested genes, ***a*** is the random polygenic effect of the mixed model, which obeys distribution $N_{n}\left(0,K\sigma_{a}^{2}\right)$, where ***K*** is the realized relationship matrix (RRM) [30,31,32,33] calculated by genetic markers and $\sigma_{a}^{2}$ is the polygenic variance, ***ε*** is a vector of errors, which follow a distribution $N_{n}\left(0,I\sigma_{\varepsilon }^{2}\right)$, where $\sigma_{\varepsilon }^{2}$ is the residual variance, **1** is a column vector of 1, ***X*** and ***Z*** are the corresponding design matrices for ***β*** and ***a***, respectively.

According to the distribution assumptions of the additive genetic and random residual effects, the adjusted phenotypic variance-covariance matrix can be described as follows:

$$\mathrm{Var}\left(y|\beta \right)=K\sigma_{a}^{2}+I\sigma_{\varepsilon }^{2}.$$

After replacing $\sigma_{a}^{2}$ with polygenic heritability $h^{2}=\sigma_{a}^{2}/\left(\sigma_{a}^{2}+\sigma_{\varepsilon }^{2}\right)$, the phenotypic variance-covariance matrix becomes:

$$\mathrm{Var}\left(y|\beta \right)=\left(\frac{h^{2}}{1-h^{2}}K+I\right)\sigma_{\varepsilon }^{2}.$$

Spectrally decompose $K=USU^{T}$ according to the FaST-LMM algorithm [20], where ***U*** and ***S*** are eigenvectors and eigenvalues of ***K*** (RRM), respectively. $U^{T}$ denotes the transpose of ***U***. As ***U*** is an orthogonal matrix ($UU^{T}=I)$, the variance-covariance matrix can be described as:

$$\mathrm{Var}\left(y|\beta \right)=U\left(\frac{h^{2}}{1-h^{2}}S+I\right)U^{T}\sigma_{\varepsilon }^{2}.$$

Let $\tilde{y}=U^{T}y$ and $\tilde{X}=U^{T}\left[1\;X\right]$, and then the LMM can be changed to a linear model (LM) in the form as follows:

$$\tilde{y}=\tilde{X}\beta +e,$$

where $e∼N_{n}\left(0,W\sigma_{\varepsilon }^{2}\right)$ with $W=\frac{h^{2}}{1-h^{2}}S+I$ being the diagonal matrix.

Using the weighted least square (WLS) or maximum likelihood (ML) estimation procedure, the parameters of ***β*** and $\sigma_{\varepsilon }^{2}$ can be estimated by maximum likelihood as:

$$\hat{\beta }={\left(\tilde{X}W^{-1}{\tilde{X}}^{T}\right)}^{-1}{\tilde{X}}^{Τ}W^{-1}\tilde{y}\;{\hat{\sigma }}_{\varepsilon }^{2}=\frac{1}{n-1}{\left(\tilde{y}-\tilde{X}\hat{\beta }\right)}^{Τ}W^{-1}(\tilde{y}-\tilde{X}\hat{\beta }).$$

Using $\hat{\beta }$ and ${\hat{\sigma }}_{\varepsilon }^{2}$, the ML function of the LM is constructed as follows:

$$L=\frac{1}{\sqrt{2\pi \left|W{\hat{\sigma }}_{\varepsilon }^{2}\right|}}\exp[\frac{1}{{\hat{\sigma }}_{\varepsilon }^{2}}(\tilde{y}-\tilde{X}\hat{\beta }{)}^{Τ}W^{-1}\left(\tilde{y}-\tilde{X}\hat{\beta }\right)].$$

To further simplify the log-likelihood value as:

$$-2\log L\propto n{\hat{\log\sigma }}_{\varepsilon }^{2}+\log|W|,$$

where the polygenic heritability h^2^ has been integrated into W. Therefore, we can optimize the log-likelihood function by using a one-dimensional scan within the open interval (0, 1) of h^2^ to obtain the maximum likelihood estimate. Meanwhile, the additive genetic effect size of the tested genes can be statistically inferred by $\hat{\beta }$ and ${\hat{\sigma }}_{\varepsilon }^{2}$ using optimized h^2^. F statistic is constructed for the gene as:

$$F=\frac{1}{df_{\beta }{\hat{\sigma }}_{\varepsilon }^{2}}\left[{\left(y-1\mu \right)}^{T}\left(y-1\mu \right)-df_{\varepsilon }{\hat{\sigma }}_{\varepsilon }^{2}\right]$$

where the degrees of freedom $df_{\beta }$ is the number of PCs screened from the tested gene and $df_{\varepsilon }=n-df_{\beta }-1$.

### 2.3. Implementation

As mentioned above, using the re-weighted least squares estimations of genes effects and optimizing polygenic heritabilities, FaST-LMM converts the whole genome mixed-model association analysis into a simple linear model association analysis. In order to improve the calculation speed, we used the fastLmPure function in the R language package RcppArmadillo to perform regression analysis on the tested genes. The calculation speed of regression analysis using fastLmPure function is faster than that of lm function. Because fastLmPure just outputs the genetic effect and standard error for the tested gene, after running the fastLmPure function, we need to additionally calculate statistics such as $\sigma_{\varepsilon }^{2}$, −2logL, student *t*-value, and *p*-value.

The input variables are obtained by converting ***X*** and ***y*** into ***X’*** and ***y’***, respectively. After polygenic heritability is given, we can get the weighted diagonal matrix ***W***. Then, the independent and dependent variables can be obtained according to ($X^{*}=W^{-\frac{1}{2}}\tilde{X}$) and ($y^{*}=W^{-\frac{1}{2}}\tilde{y}$). After preparing these variables, the process of solving LMM through barebones regression can be described as the following subroutine:
lmm <- function(ystar, xstar, w){ fit0 <- fastLmPure(y = ystar, X = as.matrix(xstar[,1])) yd <- ystar - xstar [,1]*fit0$coefficients [1] ssy <- sum(yd^2) fit <- fastLmPure(y = ystar, X = xstar) resi <- ystar-xstar%*%fit$coefficients sse <- sum(resi^2) ssr <- ssy-sse dfe <- fit$df.residual ve <- sse/dfe dfb <- ncol(xstar)-1 F <- (ssr/dfb)/ve *p* <- 1-pf(F, dfb, dfe, lower.tail = FALSE) logL<- log(det(w)) + nobs*log(ve)}

Gene heritability is the proportion of phenotypic variation explained by the tested gene. Theoretically, subtracting the heritability of the tested gene from the genomic heritability of the trait yields the polygenic heritability of the gene. Although the polygenic heritabilities of genes are different. However, because most genes do not have an effect on the quantitative trait except QTGs, the polygenic heritabilities of genes are quite close to the genomic heritability of the trait. We estimated the genomic heritability of the trait by the LMM without gene effects whose residual variance is the genetic variance. Then for the polygenic heritability of the tested gene, we can quickly find its maximum likelihood estimate by searching down from the estimate of the genomic heritability of the quantitative trait.

Substituting genome heritability for the polygenic heritability of each gene can simplify the aforementioned fast regression scan to the EMMAX algorithm [15]. The fastLmPure function is used, and the polygenic heritabilities no longer need to be optimized so that the whole genome scanning speed reaches the highest value. We can use EMMAX to estimate genetic effects and statistical probabilities as a reference for rapid regression scanning of each gene. We just select genes with a high significance level (0.05 or 0.01) or large effects in the EMMAX algorithm and optimize their polygenic heritability estimation. In this way, computational efficiency can be further improved. Therefore, the time complexity of whole genome LMM association analysis turns into O (imn), and *i* is the time spent in whole genome regression scans (1<i≤2). Relying on this, the software Single-RunKing was designed to carry out whole genome LMM association analysis on genes at a very fast speed (for the software code used for analysis, please refer to the Additional File 1 or download through the link (https://pan.baidu.com/s/1PSip3OUXOcRhnOZQynPuRQ?pwd=d3ib (accessed on 6 November 2022)).

## 3. Results

### 3.1. Simulations

**Figure 1:**
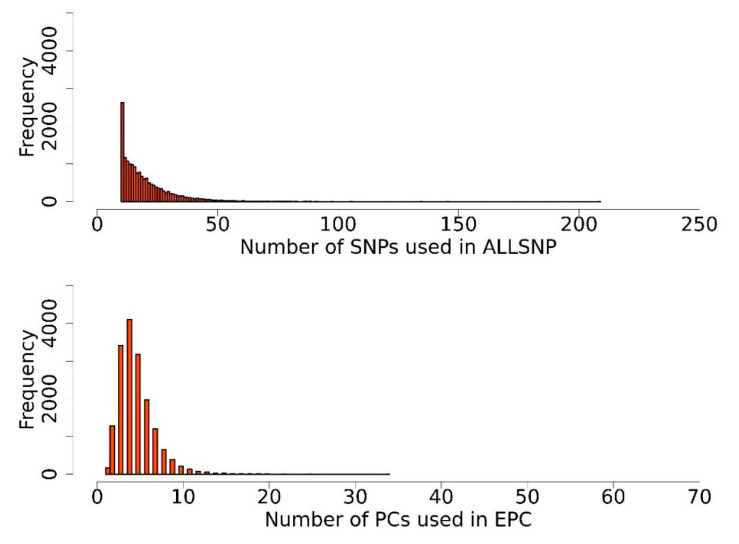
Distribution in numbers of SNPs and PCs forming gene blocks. The upper picture is the distribution in numbers of SNPs forming gene blocks by ALLSNP method. The lower picture shows the distribution in the numbers of PCs selected from each gene by EPC method.

A simulation study is conducted to investigate the statistical behavior of our proposed method using AP maize data [27]. Groups of 100, 300, 600, and 1000 QTNs were randomly assigned to the SNPs within our randomly selected 100 genes from the 16,893 genes of AP maize data, and these genes were used repeatedly in the following repeated simulations. The simulated QTNs were set to account for 60% of the phenotypic variation, and the genetic effects of these QTNs were obtained by sampling from a gamma distribution (shape = 1.66 and scale = 0.4). Next, principal component analysis was performed on the SNP markers in each gene block of the genotype matrix to make the SNP markers independent of each other. After that, each gene’s first PC and 80% PC were screened out as independent variables to represent the tested genes for GWAS analysis. Figure 1 shows the frequency of genes that use different numbers of SNPs in ALLSNP method and the frequency of genes that screen out different numbers of PCs by EPC method. The kinship matrix is constructed based on the genotype matrix composed of all SNP markers. Finally, we repeated each simulation 50 times to get the final average result.

**Figure 2:**
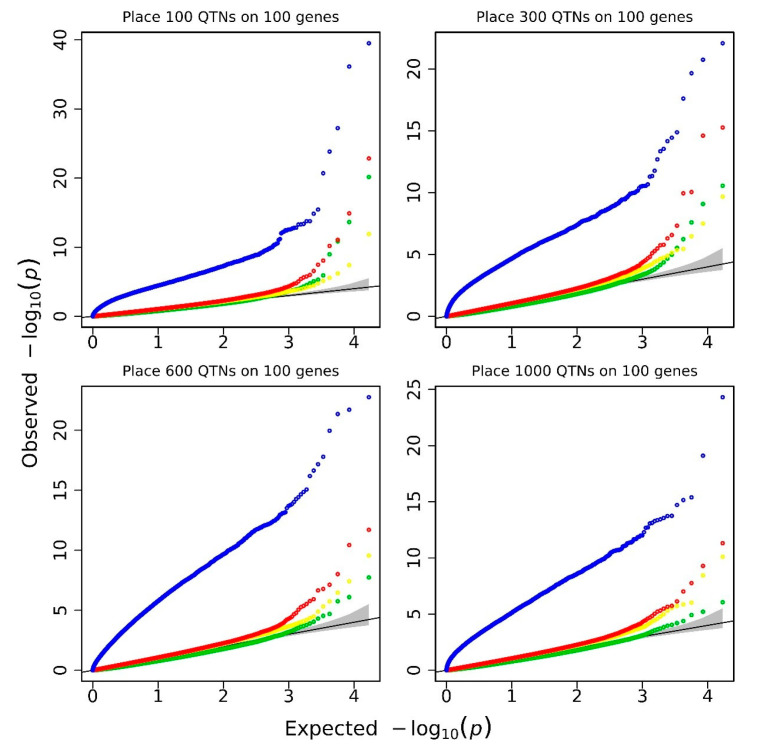
Q–Q plots for groups of 100, 300, 600, and 1000 QTNs. The green color represents ALLSNP, yellow represents FPC, red represents EPC, and blue represents EPCLM.

The simulations in this study were executed on a server with a configuration of 512 GB of RAM and 2.60-GHz Intel Xeon E5-2660 Opteron(tm) Processor, and the operating system is CentOS 6.5. ALLSNP, FPC, and EPC barebones regression scans took 24.204, 15.886, and 19.870 min, respectively, which were faster than the time taken by the linear model performed with the R/lm function (32.331, 24.353, and 31.770 min). More importantly, the statistical properties of linear mixed model methods such as EPC and FPC are much better than that of the linear model method. The false-negative/false-positive error rates were evaluated based on Q–Q plots. As shown in Figure 2, most part of the real line for −log10(*p*) obtained by the FPC and EPC method almost overlaps with the theoretical expectation, while only the high end of the line flies up due to the significant genes. This suggests that FPC and EPC exhibit good statistical properties and fit the model better than EPCLM and ALLSNP methods. EPCLM inflates test statistics rigorously, while ALLSNP deflates test statistics.

**Figure 3:**
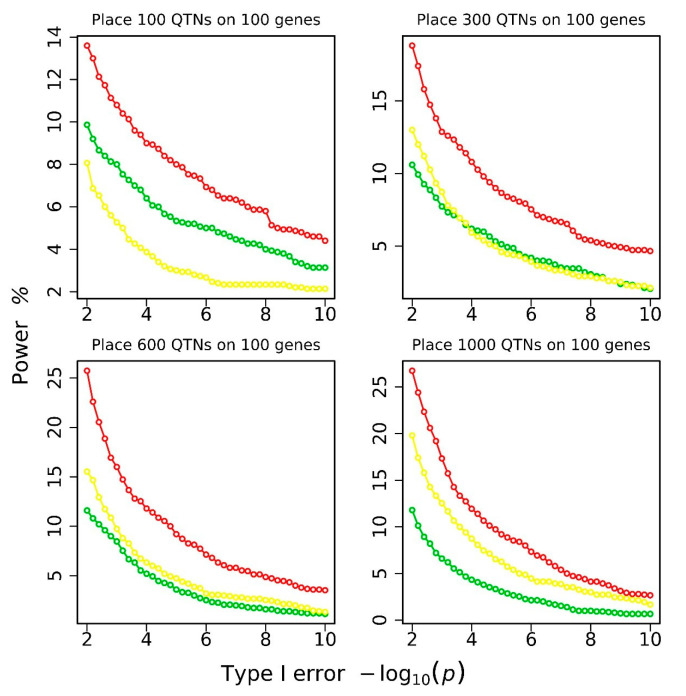
Statistical power versus different levels of type-I error for groups of 100, 300, 600, and 1000 QTNs. The green color represents ALLSNP, yellow represents FPC, red represents EPC.

A Bonferroni-corrected critical threshold of 5.529 was calculated at the 5% significance level based on the number of genes subjected to genome-wide association analysis [−log10(0.05/16,893) = 5.529]. This threshold was used to declare the significance of genes. If the tested gene passes this critical threshold and contains a pre-placed simulated QTN, then a QTG is identified. Statistical power is defined as the number of simulated QTGs identified. Statistical power plots corresponding to different type-I error levels under different QTN settings are shown in Figure 3. The power level of EPC is always higher than that of ALLSNP and FPC. No comparison to EPCLM was made because it has very high false-positive rates.

### 3.2. Case Analyses

We analyzed 20 agronomic traits of HAU maize and the days to silking (DTS) trait of AP maize [27] to evaluate the performance of the EPC method. On the same server used in the previous simulation experiments, ALLSNP, FPC, and EPC barebones regression scans took an average of 2.118, 1.159, and 1.272 min for the 20 traits in the HAU maize data, respectively. The AP dataset has more individuals than the HAU dataset, thus the running time of the three methods has increased accordingly. For the DTS trait in AP maize data, ALLSNP, FPC, and EPC barebones regression scans took 22.059, 17.052, and 19.671 min, respectively, which were faster than the time taken by the linear model performed with the R/lm function (29.545, 26.512, and 30.885 min). If only genes that pass the significance threshold of 0.05 are optimized based on EMMAX, the regression scan runtime will be reduced to 6.312, 4.901, and 5.619 min.

The Q–Q profiles of the analyzed traits are depicted on the right side of Figure 4, Figure 5 and Figures S1–S19. As shown in Table 1, for the traits analyzed above, the corresponding genomic control values (GC) for ALLSNP, FPC, EPC, and EPCLM were obtained, respectively. For example, for trait C16:0/C16:1, the GC values of methods ALLSNP, FPC, EPC, and EPCLM are 0.381, 1.017, 1.035, and 2.508, respectively. These results suggest that ALLSNP deflates test statistics significantly, whereas EPCLM significantly inflates test statistics. In addition, the ALLSNP method presents an abnormal morphology in some traits, as shown in Figure 5 and Figures S3. Compared with these two methods, methods FPC and EPC have desirable statistical properties. In the following, we would just compare the GWAS results of FPC and EPC.

**Table 1:** GC value of different methods for HAU maize and AP maize traits.

	Trait	ALLSNP	FPC	EPC	EPCLM
HAU maize	C16:0P	0.381	1.041	1.061	5.163
	C16:1P	0.397	1.032	1.044	3.557
	C18:0P	1.199	0.978	1.000	2.913
	C18:1P	0.346	1.062	1.077	4.609
	C18:2P	0.355	1.056	1.069	5.647
	C18:3P	0.312	1.074	1.073	4.808
	C20:0P	0.382	1.036	1.043	5.305
	C20:1P	0.450	1.009	1.020	3.153
	C22:0P	0.545	1.035	1.047	4.007
	C24:0P	0.340	1.047	1.059	4.378
	C16:0/C16:1	0.381	1.017	1.035	2.508
	C16:0/C18:0	0.386	1.070	1.086	3.942
	C18:0/C18:1	0.614	1.022	1.036	1.680
	C18:1/C18:2	0.466	1.028	1.049	4.508
	C18:2/C18:3	0.365	1.031	1.056	5.792
	C18:0/C20:0	0.579	1.014	1.027	3.544
	C20:0/C20:1	0.418	1.006	1.012	2.816
	C20:0/C22:0	0.410	1.050	1.070	5.315
	C22:0/C24:0	8.171	0.923	0.951	2.849
	SFA/USFA	0.373	1.035	1.055	5.384
AP maize	DTS	0.866	1.063	1.080	80.330

C16:0, palmitic acid; C16:1, palmitoleic acid; C18:0, stearic acid; C18:1, oleic acid; C18:2, linoleic acid; C18:3, linolenic acid; C20:0, arachidic acid; C20:1, gadoleic acid; C22:0, behenic acid; C24:0, lignoceric acid; SFA: Saturated fatty acid; USFA: Unsaturated fatty acid. (The suffix p represents the presence of plasmenyl group).

**Figure 4:**
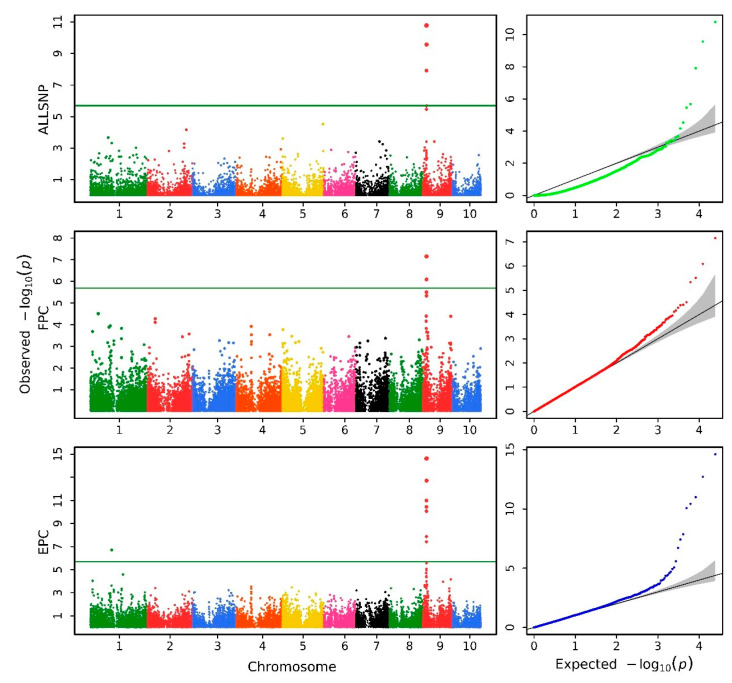
Manhattan plots (**left**) and Q–Q plots (**right**) for the C16:0/C16:1 trait of HAU maize.

**Figure 5:**
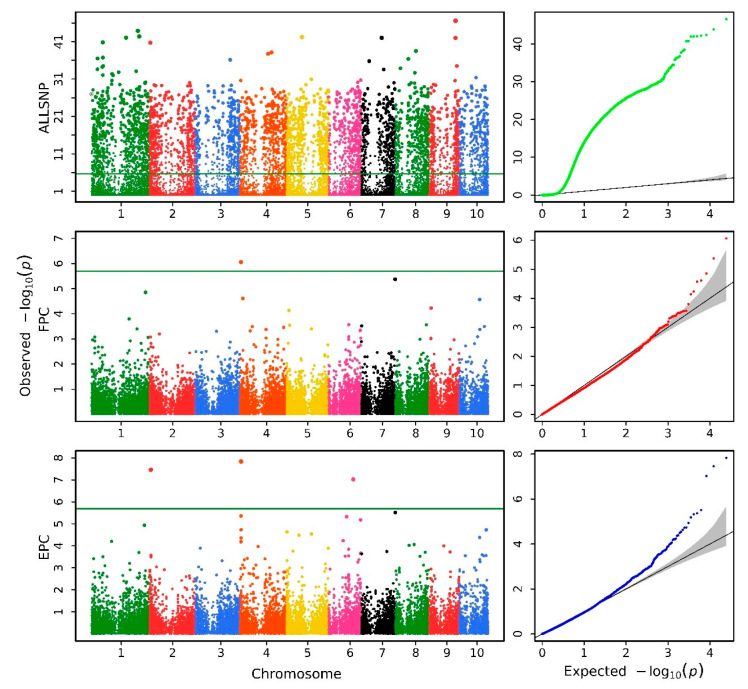
Manhattan plots (**left**) and Q–Q plots (**right**) for the C22:0/C24:0 trait of HAU maize.

As shown in Figure 4, Figure 5, and Figures S1–S19, and Tables S1 and S2, the EPC method identified more QTGs than FPC method. The horizontal reference lines represent the critical thresholds at a significance level of 5%, as obtained by Bonferroni correction. After Bonferroni correction (5.692 for HAU maize and 5.529 for AP maize), a total of 132 QTGs were detected by EPC method, while 79 QTGs were detected by FPC method, which suggests that method EPC has higher statistical efficiency. It was found that many QTGs appear in the analysis results of multiple HAU traits, which is consistent with the fact that the analyzed HAU maize traits are all traits related to kernel oil concentration and fatty acid composition. The fact that many of the same QTGs were found in the analysis of related traits also proved the reliability of our method to some extent. Finally, we reanalyzed all 34,774 genes in the HAU dataset using the EPC method (Table S3) and summarized the QTGs shared between different HAU traits (Table 2). These results provide good references for further research on pathway-based GWAS. In addition, we did a rough inspection of the genes in Table 2 and some of the most significant genes in Tables S1 and S2, and found some genes involved in fatty acid synthesis and metabolism (Table 3) according to maizeGDB website annotation. In the association analysis of the above fatty acid traits, the number of times these genes are found by the EPC method is generally much higher than that of FPC, but there are some special cases (Table 3).

## 4. Discussion

Spectral decomposition of phenotypes and markers using FaST-LMM can convert a linear mixed model into a simple linear model. We estimate the genetic effects of genes with re-weighted least square when optimizing genomic variance. The F statistic is constructed for the statistical testing of genes. In the GWAS analysis, the Single-RunKing software greatly improves the calculation efficiency mainly through three aspects: (1) Quickly estimate the genetic effects of the tested gene with LM fitting function called R/fastLmPure, (2) narrow the search to locate solutions faster by substituting heritability for genomic variance, (3) focus on large or highly significant genes captured by EMMAX. The software Single-RunKing is designed to convert whole genome mixed model analysis to simple linear model analysis. The re-weighted least square estimation of genetic effect is used to search the optimal polygenic heritability for the tested gene. Given the heritability of the genome, EMMAX requires just one round of whole genome regression scans. If only large or highly significant genes are inspected, Single-RunKing will run whole genome regression analysis based on the EMMAX method within two rounds.

Continuous advancement of re-sequencing technology will produce more high-throughput SNPs. Thus, using all the markers to construct the kinship matrix in a mixed model association study will consume increasing memory and time. In addition, if the kinship matrix changes along with the tested genes, the required computing time will be incredibly high. Moreover, computing the kinship matrix with too many SNPs could result in proximal contamination due to overestimation of polygenic variance, especially for large genetic units like genes [20,34,35]. The easiest way is to construct kinship matrices with random samples of genetic markers [16,35]. Compared with calculating the kinship matrix based on a random sample of genetic markers or all markers, selectively adding and removing pseudo-QTNs to get the kinship matrix could increase the statistical power [34,36]. In addition, in the CMLM method, individuals are divided into groups according to selected genetic markers, in this way, the dimension of RRM is reduced. For too large a resource population, genomic heritability could be estimated quickly by taking a random sample of the population. In brief, we could introduce other simplification procedures related to the whole genome mixed-model association analysis in the Single-RunKing software to enhance computational speed and power.

## 5. Conclusions

The FaST-LMM algorithm is used to convert mixed model association analysis into linear regression analysis. Gene effect and the maximum likelihood value were rapidly estimated with fastLmPure, a function for linear model fitting. When only large or highly significant genes obtained from EMMAX are tested, the extended Single-RunKing software for genes performs the entire genome regression scan within two rounds. Based on these improvements, we further compared the statistical efficiency of using 80% PC (EPC), the first PC (FPC), and all SNP markers (ALLSNP) as independent variables, which predecessors commonly use to integrate SNPs and represent genes. The EPC method fits the model well and has good statistical efficiency. It not only overcomes the false negative problem when using all SNP markers for analysis but also solves the false positive problem of EPCLM. Compared with the FPC method, it has higher statistical efficiency. The algorithm has been applied to the whole genome association study of agronomic traits in HAU and AP maize, and 132 QTGs were identified. The fact that many of the same QTGs were found in the analysis of related traits also proved the reliability of our method. Among these genes, we found some genes involved in fatty acid synthesis and metabolism according to the maizeGDB website annotation. The frequency of finding these genes by EPC method is generally much higher than that by FPC method, but there are exceptions. Therefore, we recommend using EPC for initial analysis and then using FPC as an aid to ensure foolproofness. We have been inspired that using multiple reliable and efficient methods to analyze the data simultaneously will help get better results. Different methods can be mutually validated and complementary. Finally, we observed the appearance of other significant candidate genes in multiple traits. Whether they are implicated in fatty acid synthesis and metabolism or how they influence this process is still waiting for us to explore.

## Figures and Tables

**Table 2:** QTGs shared among HAU traits.

Gene Shared among Traits	Trait Name
GRMZM2G169089	C18:0P, C18:1P, C18:2P, C18:3P, C24:0P, C16:0/C18:0, C18:1/C18:2, C20:0/C22:0
GRMZM2G169114	C18:0P, C18:1P, C18:2P, C18:3P, C24:0P, C18:1/C18:2, C20:0/C22:0
GRMZM2G173579	C16:0P, C16:0/C16:1, C16:0/C18:0, SFA/USFA
GRMZM2G173628	C16:0P, C16:0/C16:1, C16:0/C18:0, SFA/USFA
GRMZM2G064701	C18:1P, C18:2P, C18:0/C18:1, C18:1/C18:2
GRMZM2G173615	C16:0P, C16:0/C16:1, SFA/USFA
GRMZM2G173641	C16:0P, C16:0/C16:1, SFA/USFA
GRMZM2G444801	C16:0P, C16:0/C16:1, SFA/USFA
GRMZM5G829544	C16:0P, C16:0/C16:1, SFA/USFA
GRMZM5G867927	C18:1P, C18:2P, C18:0/C18:1, C18:1/C18:2
GRMZM2G029506	C20:0P, C22:0P, C24:0P
GRMZM2G125455	C18:1P, C18:2P, C18:1/C18:2
GRMZM2G125544	C18:1P, C18:2P
GRMZM2G149138	C18:1P, C18:2P, C18:1/C18:2
GRMZM2G173678	C16:0P, C16:0/C16:1, SFA/USFA
GRMZM2G335618	C20:0P, C22:0P, C18:0/C20:0
GRMZM2G365292	C18:1P, C18:2P, C18:1/C18:2
GRMZM2G444623	C18:1P, C18:2P, C18:1/C18:2
GRMZM2G449817	C22:0P, C24:0P, C20:0/C22:0
GRMZM2G005339	C16:0P, SFA/USFA
GRMZM2G075637	C16:0P, C16:0/C18:0
GRMZM2G094871	C18:1P, C18:1/C18:2
GRMZM2G101707	C20:1P, C22:0P
GRMZM2G103475	C16:0P, SFA/USFA
GRMZM2G109009	C18:2P, C20:0/C22:0
GRMZM2G404897	C16:0P, SFA/USFA
GRMZM2G461671	C18:1P, C18:1/C18:2
GRMZM5G899300	C16:0P, SFA/USFA

**Table 3:** Genes involved in fatty acid synthesis and metabolism.

Gene	Function and Pathway	Number of Times Found by FPC	Number of Times Found by EPC
GRMZM2G169089	linoleic acid1, triacylglycerol biosynthesis pathway	9	8
GRMZM2G064701	fatty acid desaturase1	3	4
GRMZM5G829544	fatty acyl-ACP thioesterase2, oleate biosynthesis I (plants)	0	4
GRMZM5G867927	fatty acid desaturase1	3	4
GRMZM2G022558	fatty acid elongase2	2	1
GRMZM2G370357	lipid metabolic process	Not Available	1 (From Table S3)

## Data Availability

The HAU maize datasets used in this study are publicly available at the website of Jianbing Yan (http://www.maizego.org/Resources.html (accessed on 6 November 2022)) (https://doi.org/10.1016/j.molp.2016.06.016), or through the direct download link (https://pan.baidu.com/s/1gQbyHq01LNeOxE3hU8ZIzA?pwd=8vuc (accessed on 6 November 2022)). The AP maize datasets analyzed in the current study are free downloaded from website (http://www.panzea.org/#!genotypes/cctl (accessed on 6 November 2022)) (DOI: 10.1186/gb-2013-14-6-r55), or through the direct download link (https://pan.baidu.com/s/1bIt_a3DAkbDfLCGx41mv2w?pwd=awvy (accessed on 6 November 2022)). For the software code used for analysis, please refer to the Supplementary File S1 or download through the link (https://pan.baidu.com/s/1PSip3OUXOcRhnOZQynPuRQ?pwd=d3ib (accessed on 6 November 2022)).

## Supplementary Materials

The following supporting information can be downloaded at: https://www.mdpi.com/article/10.3390/biology11111649/s1, Figure S1: Manhattan plots (left) and Q–Q plots (right) for the C16:0P trait of HAU maize; Figure S2: Manhattan plots (left) and Q–Q plots (right) for the C16:1P trait of maize; Figure S3: Manhattan plots (left) and Q–Q plots (right) for the C18:0P trait of maize; Figure S4: Manhattan plots (left) and Q–Q plots (right) for the C18:1P trait of maize; Figure S5: Manhattan plots (left) and Q–Q plots (right) for the C18:2P trait of maize; Figure S6: Manhattan plots (left) and Q–Q plots (right) for the C18:3P trait of maize; Figure S7: Manhattan plots (left) and Q–Q plots (right) for the C20:0P trait of maize; Figure S8: Manhattan plots (left) and Q–Q plots (right) for the C20:1P trait of maize; Figure S9: Manhattan plots (left) and Q–Q plots (right) for the C22:0P trait of maize; Figure S10: Manhattan plots (left) and Q–Q plots (right) for the C24:0P trait of maize; Figure S11: Manhattan plots (left) and Q–Q plots (right) for the C16:0/C18:0 trait of maize; Figure S12: Manhattan plots (left) and Q–Q plots (right) for the C18:0/C18:1 trait of maize; Figure S13: Manhattan plots (left) and Q–Q plots (right) for the C18:1/C18:2 trait of maize; Figure S14: Manhattan plots (left) and Q–Q plots (right) for the C18:2/C18:3 trait of maize; Figure S15: Manhattan plots (left) and Q–Q plots (right) for the C18:0/C20:0 trait of maize; Figure S16: Manhattan plots (left) and Q–Q plots (right) for the C20:0/C20:1 trait of maize; Figure S17: Manhattan plots (left) and Q–Q plots (right) for the C20:0/C22:0 trait of maize; Figure S18: Manhattan plots (left) and Q–Q plots (right) for the SFA/USFA trait of maize; Figure S19: Manhattan plots (left) and Q–Q plots (right) for the DTS trait of AP maize; Table S1: QTGs identified for 20 HAU maize traits and one AP maize trait with EPC method; Table S2: QTGs identified for 20 HAU maize traits and one AP maize trait with FPC method; Table S3: QTGs identified for 20 HAU maize traits and one AP maize trait with EPC method (From analysis of all the 34,774 HAU genes).

## Abbreviations

ALLSNP, the linear mixed model method that using all SNPs belonging to each gene as the independent variable; AP, Ames panel; DTS, Days to silking; EPC, the linear mixed model method that using 80% PCs belonging to each gene as the independent variable; EPCLM, the simple linear model method that using 80% PCs belonging to each gene as the independent variable; FPC, the linear mixed model method that using the first PC belonging to each gene as the independent variable; FaST-LMM, Factored spectrally transformed linear mixed model; HAU, Huazhong Agricultural University; LM, linear model; LMM, linear mixed model; PC, principal component; QTG, quantitative trait genes; RRM, Realized relationship matrix.
