# Supplementary material for: Gene-Based Genome-Wide Association Study Identified Genes for Agronomic Traits in Maize
Source: Biology (Basel). 2022 Nov 11;11(11):1649. doi: 10.3390/biology11111649 (PMC9687540; doi:10.3390/biology11111649)
Supplement: Supplementary file 1 [file biology-11-01649-s001.zip › Supplementary File S1.pdf]

Supplementary Material

Supplementary Tables

Table S1 QTGs identified for 20 HAU maize traits and one AP maize trait with EPC method

Trait	Gene ID	Chr	Position(bp)	$-\log_{10}(p)$
C16:0P	GRMZM2G075637	5	176387128-176391570	6.344
	GRMZM2G005339	9	19290570-19292460	6.883
	GRMZM2G103475	9	20605661-20607692	8.896
	GRMZM2G146490	9	20431547-20438473	5.715
	GRMZM2G173615	9	20470860-20471877	13.863
	GRMZM2G173628	9	20467340-20469518	20.589
	GRMZM2G173641	9	20462059-20467072	11.919
	GRMZM2G173678	9	20462041-20464497	9.249
	GRMZM2G404897	9	20735631-20746313	7.678
	GRMZM2G444801	9	20333819-20338856	10.649
	GRMZM5G829544	9	20583726-20587950	21.888
	GRMZM5G899300	9	20597214-20603345	9.211
C16:1P	GRMZM2G015767	1	112726894-112734589	7.367
	GRMZM2G052650	1	170847950-170852831	5.855
	GRMZM2G171713	1	150897326-150899612	5.735
C18:0P	GRMZM2G179454	5	92183288-92186615	6.692
	GRMZM2G169089	6	104859135-104866019	6.272
	GRMZM2G169114	6	104848940-104858629	7.076
C18:1P	GRMZM2G061303	4	162342026-162344517	5.826
	GRMZM2G064145	4	117263768-117268901	5.734
	GRMZM2G064701	4	162256197-162265080	14.500
	GRMZM2G094871	4	118897364-118900639	6.118
	GRMZM2G125455	4	166000595-166004995	11.905
	GRMZM2G125544	4	166009200-166014070	6.855
	GRMZM2G137413	4	118695002-118700985	5.980
	GRMZM2G149138	4	119701209-119703693	6.222
	GRMZM2G365292	4	162264226-162264976	7.687
	GRMZM2G444623	4	166480988-166482727	7.133
	GRMZM5G867927	4	162256559-162257902	11.754
	GRMZM2G169089	6	104859135-104866019	16.217
	GRMZM2G169114	6	104848940-104858629	13.606
C18:2P	GRMZM2G064701	4	162256197-162265080	13.364
	GRMZM2G125455	4	166000595-166004995	9.708
	GRMZM2G125544	4	166009200-166014070	6.234
	GRMZM2G149138	4	119701209-119703693	6.153
	GRMZM2G365292	4	162264226-162264976	7.519
	GRMZM2G444623	4	166480988-166482727	6.493
	GRMZM5G867927	4	162256559-162257902	10.423
	GRMZM2G109009	6	104715306-104721588	6.670
	GRMZM2G169089	6	104859135-104866019	16.423
	GRMZM2G169114	6	104848940-104858629	13.062

C18:3P	GRMZM2G169089	6	104859135-104866019	10.546
	GRMZM2G169114	6	104848940-104858629	9.963
C20:0P	GRMZM2G029506	6	104976057-104986465	7.024
	GRMZM2G032028	6	103866328-103869085	6.856
	GRMZM2G064518	6	104269642-104276065	6.524
	GRMZM2G070378	6	106095389-106156944	6.089
	GRMZM2G071638	6	106176163-106181370	6.629
	GRMZM2G129431	6	106338056-106351289	8.986
	GRMZM2G129783	6	106466750-106469829	6.655
	GRMZM2G138842	6	111021704-111031563	7.870
	GRMZM2G139029	6	110807326-110809938	5.735
	GRMZM2G148460	6	106066972-106069744	6.044
	GRMZM2G162295	6	103986649-103989006	5.838
	GRMZM2G335618	6	103857605-103858686	6.451
	GRMZM2G335635	6	103867383-103867951	6.812
	GRMZM2G442244	6	111053353-111057384	7.968
C20:1P	GRMZM2G031790	1	12944095-12947312	6.106
	GRMZM2G101707	6	104454744-104456719	5.888
C22:0P	GRMZM2G029506	6	104976057-104986465	6.782
	GRMZM2G029685	6	104965877-104967497	6.769
	GRMZM2G101707	6	104454744-104456719	6.034
	GRMZM2G169114	6	104848940-104858629	5.732
	GRMZM2G335618	6	103857605-103858686	8.441
	GRMZM2G449817	6	104671217-104674448	7.060
C24:0P	GRMZM2G029506	6	104976057-104986465	6.758
	GRMZM2G169089	6	104859135-104866019	12.043
	GRMZM2G169114	6	104848940-104858629	11.982
	GRMZM2G449817	6	104671217-104674448	7.977
C16:0/C16:1	GRMZM2G002515	1	113527662-113531214	6.718
	GRMZM2G146490	9	20431547-20438473	7.415
	GRMZM2G173615	9	20470860-20471877	10.993
	GRMZM2G173628	9	20467340-20469518	12.712
	GRMZM2G173641	9	20462059-20467072	10.434
	GRMZM2G173678	9	20462041-20464497	7.867
	GRMZM2G444801	9	20333819-20338856	10.066
	GRMZM5G829544	9	20583726-20587950	14.623
C16:0/C18:0	GRMZM2G075637	5	176387128-176391570	7.217
	AC202439.3_FG006	6	113667684-113673250	6.159
	GRMZM2G169089	6	104859135-104866019	5.913
	GRMZM2G173628	9	20467340-20469518	6.803
	GRMZM5G829544	9	20583726-20587950	5.759
C18:0/C18:1	GRMZM2G064701	4	162256197-162265080	7.531
	GRMZM5G867927	4	162256559-162257902	6.038
C18:1/C18:2	GRMZM2G064701	4	162256197-162265080	11.983

	GRMZM2G094871	4	118897364-118900639	5.896
	GRMZM2G125455	4	166000595-166004995	10.173
	GRMZM2G137413	4	118695002-118700985	5.834
	GRMZM2G149138	4	119701209-119703693	6.485
	GRMZM2G365292	4	162264226-162264976	6.694
	GRMZM2G444623	4	166480988-166482727	6.725
	GRMZM2G453424	4	162903711-162910164	5.793
	GRMZM5G867927	4	162256559-162257902	9.847
	GRMZM2G169089	6	104859135-104866019	13.503
	GRMZM2G169114	6	104848940-104858629	11.267
C18:2/C18:3	GRMZM2G015080	2	150143387-150147208	6.149
	GRMZM5G874168	3	178136492-178137419	5.740
C18:0/C20:0	GRMZM2G335618	6	103857605-103858686	5.951
	GRMZM2G004128	9	86529826-86531722	8.701
	GRMZM2G022275	9	86866692-86874101	8.303
	GRMZM2G022558	9	86863072-86865460	10.777
	GRMZM2G148872	9	86665644-86670790	11.101
	GRMZM2G153541	9	87061673-87064972	10.603
C20:0/C20:1				
C20:0/C22:0	GRMZM2G048435	5	26660015-26688602	6.418
	GRMZM2G048559	5	26654931-26658378	6.007
	GRMZM2G173978	5	26765606-26775498	6.603
	GRMZM2G109009	6	104715306-104721588	7.290
	GRMZM2G169089	6	104859135-104866019	29.832
	GRMZM2G169114	6	104848940-104858629	29.617
	GRMZM2G383234	6	103794573-103795230	6.386
	GRMZM2G449817	6	104671217-104674448	6.968
	GRMZM5G884556	6	103794595-103795566	5.813
	GRMZM2G003022	8	38520650-38525237	7.265
	GRMZM2G097103	8	38489499-38493433	7.008
C22:0/C24:0	GRMZM5G848138	2	6556394-6560322	7.458
	GRMZM2G346897	4	5140555-5141628	7.832
	GRMZM2G165865	6	128470605-128471283	7.022
SFA/USFA	GRMZM2G005339	9	19290570-19292460	6.040
	GRMZM2G103475	9	20605661-20607692	6.774
	GRMZM2G173615	9	20470860-20471877	10.600
	GRMZM2G173628	9	20467340-20469518	15.939
	GRMZM2G173641	9	20462059-20467072	8.961
	GRMZM2G173678	9	20462041-20464497	7.068
	GRMZM2G404897	9	20735631-20746313	5.949
	GRMZM2G444801	9	20333819-20338856	7.491
	GRMZM5G829544	9	20583726-20587950	16.142
	GRMZM5G899300	9	20597214-20603345	7.210
DTS	GRMZM2G005163	8	133187005-133198530	7.122

	GRMZM2G053991	8	132371470-132373148	6.946
	GRMZM2G075000	8	133259612-133261225	6.510
	GRMZM2G102674	8	133275401-133277844	7.839
	GRMZM2G120839	8	131174771-131178117	5.799
	GRMZM2G140083	8	132327747-132328904	8.471
	GRMZM2G173763	8	132200869-132205133	7.719
	GRMZM2G479987	8	123509007-123513267	9.808
	GRMZM2G700665	8	132044001-132047428	9.144

Table S2 QTGs identified for 20 HAU maize traits and one AP maize trait with FPC method

Trait	Gene ID	Chr	Position (bp)	$-\log_{10}(p)$
C16:0P	GRMZM2G005339	9	19290570-19292460	7.558
	GRMZM2G146490	9	20431547-20438473	6.888
	GRMZM2G173615	9	20470860-20471877	11.066
	GRMZM2G173693	9	20451756-20455841	6.352
	GRMZM5G899300	9	20597214-20603345	6.258
C16:1P				
C18:0P	GRMZM2G169089	6	104859135-104866019	7.302
	GRMZM2G169114	6	104848940-104858629	7.518
C18:1P	GRMZM2G055158	4	166910417-166911806	6.448
	GRMZM2G061303	4	162342026-162344517	7.512
	GRMZM2G064145	4	117263768-117268901	5.912
	GRMZM2G064701	4	162256197-162265080	7.096
	GRMZM2G125544	4	166009200-166014070	6.853
	GRMZM2G365292	4	162264226-162264976	7.175
	GRMZM2G444623	4	166480988-166482727	6.153
	GRMZM5G867927	4	162256559-162257902	11.036
	GRMZM2G169089	6	104859135-104866019	17.592
	GRMZM2G169114	6	104848940-104858629	14.195
C18:2P	GRMZM2G055158	4	166910417-166911806	5.977
	GRMZM2G061303	4	162342026-162344517	7.064
	GRMZM2G064701	4	162256197-162265080	6.613
	GRMZM2G064960	4	162265417-162267773	6.324
	GRMZM2G125544	4	166009200-166014070	6.725
	GRMZM2G365292	4	162264226-162264976	7.575
	GRMZM2G444623	4	166480988-166482727	5.878
	GRMZM5G867927	4	162256559-162257902	10.004
	GRMZM2G169089	6	104859135-104866019	17.924
	GRMZM2G169114	6	104848940-104858629	13.590
	GRMZM2G383234	6	103794573-103795230	5.856
	GRMZM5G884556	6	103794595-103795566	5.908
C18:3P	GRMZM2G169089	6	104859135-104866019	11.605
	GRMZM2G169114	6	104848940-104858629	10.737
C20:0P	GRMZM2G032028	6	103866328-103869085	7.972
	GRMZM2G101938	6	105424578-105432613	6.588
	GRMZM2G103526	6	104755709-104760627	5.935
	GRMZM2G129783	6	106466750-106469829	7.791
	GRMZM2G138842	6	111021704-111031563	6.683
	GRMZM2G335635	6	103867383-103867951	7.748
C20:1P	GRMZM2G041809	4	181870770-181873108	5.762
	GRMZM2G103526	6	104755709-104760627	6.146
	GRMZM2G449817	6	104671217-104674448	6.389

C22:0P	GRMZM2G169089	6	104859135-104866019	6.190
	GRMZM2G169114	6	104848940-104858629	6.246
C24:0P	GRMZM2G169089	6	104859135-104866019	13.609
	GRMZM2G169114	6	104848940-104858629	12.408
C16:0/C16:1	GRMZM2G173615	9	20470860-20471877	7.156
	GRMZM2G173693	9	20451756-20455841	6.092
C16:0/C18:0	GRMZM2G169089	6	104859135-104866019	6.903
	GRMZM2G169114	6	104848940-104858629	6.115
C18:0/C18:1	GRMZM2G022558	9	86863072-86865460	6.026
C18:1/C18:2	GRMZM2G061303	4	162342026-162344517	6.274
	GRMZM2G064701	4	162256197-162265080	5.702
	GRMZM2G365292	4	162264226-162264976	6.298
	GRMZM5G867927	4	162256559-162257902	9.033
	GRMZM2G169089	6	104859135-104866019	14.703
	GRMZM2G169114	6	104848940-104858629	11.904
C18:2/C18:3				
C18:0/C20:0	GRMZM2G022275	9	86866692-86874101	9.187
	GRMZM2G022558	9	86863072-86865460	11.297
	GRMZM2G148872	9	86665644-86670790	8.821
	GRMZM2G153541	9	87061673-87064972	11.147
C20:0/C20:1	GRMZM2G376905	4	80929048-80934098	5.731
C20:0/C22:0	GRMZM2G173978	5	26765606-26775498	6.704
	GRMZM2G169089	6	104859135-104866019	30.453
	GRMZM2G169114	6	104848940-104858629	30.101
	GRMZM2G383234	6	103794573-103795230	6.359
	GRMZM5G884556	6	103794595-103795566	6.556
C22:0/C24:0	GRMZM2G045387	4	5122891-5123921	6.061
SFA/USFA	GRMZM2G005339	9	19290570-19292460	6.765
	GRMZM2G173615	9	20470860-20471877	8.922
DTS	AC214648.3_FG001	8	131903551-131905086	5.743
	GRMZM2G005163	8	133187005-133198530	6.696
	GRMZM2G053991	8	132371470-132373148	8.866
	GRMZM2G075000	8	133259612-133261225	6.950
	GRMZM2G102674	8	133275401-133277844	8.420
	GRMZM2G120839	8	131174771-131178117	6.344
	GRMZM2G140083	8	132327747-132328904	6.804
	GRMZM2G173763	8	132200869-132205133	8.907
	GRMZM2G353250	8	132372125-132373163	6.555
	GRMZM2G479987	8	123509007-123513267	6.880
	GRMZM2G700665	8	132044001-132047428	6.820

Table S3 QTGs identified for 20 HAU maize traits and one AP maize trait with EPC method (From analysis of all the 34774 HAU genes)

Trait	Gene ID	Chr	Position (bp)	$-\log_{10}(p)$
C16:0P	GRMZM2G084249	1	185221195-185222027	6.105
	GRMZM2G075637	5	176387128-176391570	6.307
	GRMZM2G005339	9	19290570-19292460	6.844
	GRMZM2G103475	9	20605661-20607692	8.878
	GRMZM2G173579	9	20473674-20477553	19.140
	GRMZM2G173615	9	20470860-20471877	13.848
	GRMZM2G173628	9	20467340-20469518	20.558
	GRMZM2G173641	9	20462059-20467072	11.908
	GRMZM2G173678	9	20462041-20464497	9.243
	GRMZM2G404897	9	20735631-20746313	7.690
	GRMZM2G444801	9	20333819-20338856	10.696
	GRMZM5G829544	9	20583726-20587950	21.911
	GRMZM5G899300	9	20597214-20603345	9.217
C16:1P	GRMZM2G015767	1	112726894-112734589	7.327
	GRMZM2G052650	1	170847950-170852831	5.913
C18:0P	GRMZM2G086346	2	145355612-145357242	7.899
	GRMZM2G179454	5	92183288-92186615	6.754
	GRMZM2G396169	5	115390945-115402249	6.122
	GRMZM2G169089	6	104859135-104866019	6.262
	GRMZM2G169114	6	104848940-104858629	7.092
	GRMZM5G822819	6	13386458-13387027	7.226
C18:1P	GRMZM2G064701	4	162256197-162265080	14.444
	GRMZM2G094871	4	118897364-118900639	6.110
	GRMZM2G125455	4	166000595-166004995	11.865
	GRMZM2G125544	4	166009200-166014070	6.880
	GRMZM2G137413	4	118695002-118700985	5.972
	GRMZM2G149138	4	119701209-119703693	6.208
	GRMZM2G365292	4	162264226-162264976	7.656
	GRMZM2G444623	4	166480988-166482727	7.201
	GRMZM2G461671	4	166346782-166348531	6.438
	GRMZM5G867927	4	162256559-162257902	11.700
	GRMZM2G169089	6	104859135-104866019	16.258
	GRMZM2G169114	6	104848940-104858629	13.627
C18:2P	GRMZM2G064701	4	162256197-162265080	13.323
	GRMZM2G125455	4	166000595-166004995	9.681
	GRMZM2G125544	4	166009200-166014070	6.248
	GRMZM2G149138	4	119701209-119703693	6.162
	GRMZM2G365292	4	162264226-162264976	7.493
	GRMZM2G444623	4	166480988-166482727	6.547
	GRMZM5G867927	4	162256559-162257902	10.391
	GRMZM2G109009	6	104715306-104721588	6.721

	GRMZM2G169089	6	104859135-104866019	16.446
	GRMZM2G169114	6	104848940-104858629	13.085
C18:3P	GRMZM2G169089	6	104859135-104866019	10.638
	GRMZM2G169114	6	104848940-104858629	10.044
C20:0P	GRMZM2G029506	6	104976057-104986465	7.030
	GRMZM2G032028	6	103866328-103869085	6.868
	GRMZM2G064518	6	104269642-104276065	6.482
	GRMZM2G070378	6	106095389-106156944	6.062
	GRMZM2G071638	6	106176163-106181370	6.635
	GRMZM2G129431	6	106338056-106351289	8.993
	GRMZM2G129783	6	106466750-106469829	6.646
	GRMZM2G138842	6	111021704-111031563	7.885
	GRMZM2G148460	6	106066972-106069744	6.032
	GRMZM2G335618	6	103857605-103858686	6.408
	GRMZM2G335635	6	103867383-103867951	6.820
	GRMZM2G442244	6	111053353-111057384	7.985
C20:1P	GRMZM2G031790	1	12944095-12947312	6.184
	GRMZM2G101707	6	104454744-104456719	5.885
C22:0P	GRMZM2G029506	6	104976057-104986465	6.790
	GRMZM2G029685	6	104965877-104967497	6.768
	GRMZM2G101707	6	104454744-104456719	6.034
	GRMZM2G335618	6	103857605-103858686	8.389
	GRMZM2G449817	6	104671217-104674448	7.013
C24:0P	GRMZM2G152793	5	210159498-210162293	6.176
	GRMZM2G029506	6	104976057-104986465	6.792
	GRMZM2G169089	6	104859135-104866019	12.123
	GRMZM2G169114	6	104848940-104858629	12.062
	GRMZM2G449817	6	104671217-104674448	8.006
C16:0/C16:1	GRMZM2G002515	1	113527662-113531214	6.670
	GRMZM2G146490	9	20431547-20438473	7.449
	GRMZM2G173579	9	20473674-20477553	11.684
	GRMZM2G173615	9	20470860-20471877	11.019
	GRMZM2G173628	9	20467340-20469518	12.763
	GRMZM2G173641	9	20462059-20467072	10.463
	GRMZM2G173678	9	20462041-20464497	7.895
	GRMZM2G444801	9	20333819-20338856	10.070
	GRMZM5G829544	9	20583726-20587950	14.623
C16:0/C18:0	GRMZM2G075637	5	176387128-176391570	7.194
	AC202439.3_FG006	6	113667684-113673250	6.094
	GRMZM2G169089	6	104859135-104866019	5.912
	GRMZM2G173579	9	20473674-20477553	7.674
	GRMZM2G173628	9	20467340-20469518	6.756
C18:0/C18:1	GRMZM2G064701	4	162256197-162265080	7.525
	GRMZM2G081285	4	156969964-156970836	6.599

	GRMZM5G867927	4	162256559-162257902	6.000
C18:1/C18:2	GRMZM2G064701	4	162256197-162265080	11.920
	GRMZM2G094871	4	118897364-118900639	5.893
	GRMZM2G125455	4	166000595-166004995	10.128
	GRMZM2G149138	4	119701209-119703693	6.476
	GRMZM2G365292	4	162264226-162264976	6.659
	GRMZM2G444623	4	166480988-166482727	6.788
	GRMZM2G461671	4	166346782-166348531	5.853
	GRMZM5G867927	4	162256559-162257902	9.796
	GRMZM2G169089	6	104859135-104866019	13.544
	GRMZM2G169114	6	104848940-104858629	11.292
C18:2/C18:3	GRMZM2G024374	1	298324391-298326544	6.039
	GRMZM2G015080	2	150143387-150147208	6.195
	GRMZM2G095366	8	24868167-24869609	6.590
C18:0/C20:0	GRMZM2G335618	6	103857605-103858686	5.916
	GRMZM2G004128	9	86529826-86531722	8.764
	GRMZM2G022275	9	86866692-86874101	8.339
	GRMZM2G022558	9	86863072-86865460	10.880
	GRMZM2G148872	9	86665644-86670790	11.199
	GRMZM2G153541	9	87061673-87064972	10.668
C20:0/C20:1				
C20:0/C22:0	GRMZM2G048435	5	26660015-26688602	6.489
	GRMZM2G048559	5	26654931-26658378	6.075
	GRMZM2G173978	5	26765606-26775498	6.678
	GRMZM2G109009	6	104715306-104721588	7.299
	GRMZM2G169089	6	104859135-104866019	29.891
	GRMZM2G169114	6	104848940-104858629	29.705
	GRMZM2G383234	6	103794573-103795230	6.434
	GRMZM2G449817	6	104671217-104674448	6.921
	GRMZM5G884556	6	103794595-103795566	5.863
	GRMZM2G003022	8	38520650-38525237	7.335
	GRMZM2G097103	8	38489499-38493433	7.059
C22:0/C24:0	GRMZM2G158434	1	205416591-205417331	7.856
	GRMZM2G561630	1	277377077-277377180	8.698
	GRMZM2G564571	2	229943034-229943935	6.983
	GRMZM5G848138	2	6556394-6560322	7.483
	AC197030.3_FG002	3	73135390-73137082	8.922
	GRMZM2G044625	4	5117703-5118736	8.601
	GRMZM2G160143	4	83173193-83174466	6.152
	GRMZM2G160268	4	83173196-83174639	6.152
	GRMZM2G174255	4	139412421-139413949	5.997
	GRMZM2G346897	4	5140555-5141628	7.837
	GRMZM2G459828	4	40010831-40016436	7.807
	GRMZM5G827945	4	18916255-18917109	7.546

	GRMZM2G040871	5	64625580-64628249	8.447
	GRMZM2G325653	5	64674608-64681233	9.281
	GRMZM2G370357	5	43156300-43159749	19.427
	AC200287.4_FG006	6	86042427-86047290	8.874
	GRMZM2G165865	6	128470605-128471283	7.219
	GRMZM2G166956	10	110833467-110837705	6.745
	GRMZM2G305757	10	60798934-60799604	7.286
SFA/USFA	GRMZM2G005339	9	19290570-19292460	6.014
	GRMZM2G103475	9	20605661-20607692	6.764
	GRMZM2G173579	9	20473674-20477553	14.724
	GRMZM2G173615	9	20470860-20471877	10.609
	GRMZM2G173628	9	20467340-20469518	15.929
	GRMZM2G173641	9	20462059-20467072	8.963
	GRMZM2G173678	9	20462041-20464497	7.071
	GRMZM2G404897	9	20735631-20746313	5.981
	GRMZM2G444801	9	20333819-20338856	7.545
	GRMZM5G829544	9	20583726-20587950	16.163
	GRMZM5G899300	9	20597214-20603345	7.213

Program for Single-Runking

```
####################### subroutine ########################
GBLUP <- function(ynew,gnew){
	nobs <- nrow(gnew)
	pre_loglike <- 10^10
	ynew <- V050*ynew[,1]
	for(i in 1:length(hh)){
		gnew1 <- V050[,i]*gnew
		fit=fastLmPure(y = ynew[,i], X = gnew1)
		eff <- fit$coefficients
		resi <- ynew[,i]-gnew1%*%as.matrix(eff)
		ve <- sum(resi^2)/(nobs-ncol(gnew)-1)
		loglike <- logV0[i]+nobs*log(ve)
		if(loglike > pre_loglike){
			break
		}else{
			pre_eff <- eff
			h2 <- hh[i]
			pre_ve <- ve
			pre_loglike <- loglike
			}
	}
	fva <- list(eff=pre_eff,h2=h2,ve=pre_ve,loglike=pre_loglike)
	return(c(fva))
}

fast_lmm_block <- function(ynew1,gnew,threshold0){
	gnew1 <- V050[,hpos]*gnew
	f10 <- fastlm_block(hpos,ynew1,gnew1)
	if(f10[2] < threshold0){
		pre_eff <- f10[1]
		pre_p <- f10[2]
		pre_loglike <- f10[3]
		pre_F <- f10[4]
		for(j in 1:(hpos-1)){
			i <- hpos-j
			gnew1 <- V050[,i]*gnew
			f10 <- fastlm_block(i,ynew1,gnew1)
			loglike <- f10[3]
			if(loglike > pre_loglike) break
			if(i==1) cat("No Heritability!","\n")
			pre_loglike <- loglike
			pre_eff <- f10[1]
			pre_p <- f10[2]
			pre_F <- f10[4]
		}
		hhk <- hh[i+1]
		f10 <- c(pre_eff,pre_p,pre_loglike,pre_F)
```

```
			if(hhk==hh0){
				pre_eff <- f10[1]
				pre_p <- f10[2]
				pre_loglike <- f10[3]
				pre_F <- f10[4]
				for(j in 1:(length(hh)-hpos)){
					i <- hpos+j
					gnew1 <- V050[,i]*gnew
					f10 <- fastlm_block(i,ynew1,gnew1)
					loglike <- f10[3]
					if(loglike > pre_loglike) break
					if(i==length(hh)) cat("No Solution!","\n")
					pre_loglike <- loglike
					pre_eff <- f10[1]
					pre_p <- f10[2]
					pre_F <- f10[4]
				}
				f10 <- c(pre_eff,pre_p,pre_loglike,pre_F)
			}
  }
	return(f10)
}

fastlm_block <- function(i,ynew1,gnew1){
			Fs <- c(rep(0,3))
			fit0 <- fastLmPure(y = ynew1[,i], X = as.matrix(gnew1[,1]))
			y0 <- ynew1[,i]-gnew1[,1]*fit0$coefficients[1]
			ssy <- sum(y0^2)
			fit <- fastLmPure(y = ynew1[,i], X = gnew1)
			resi <- ynew1[,i]-gnew1%*%fit$coefficients
			sse <- sum(resi^2)
			ssr <- ssy-sse
			Fs[3] <- fit$df.residual
			ve <- sse/Fs[3]
			Fs[2] <- ncol(gnew1)-1
			Fs[1] <- (ssr/Fs[2])/ve
			p <- pf(Fs[1],Fs[2],Fs[3],lower.tail = FALSE)
			loglike0 <- logV0[i]+nobs*log(ve)
			fastlm_block <- c(fit$coefficients[1],p,loglike0,Fs[1])
}

QQ_plot <- function(methodname,pValue){
	jpeg(file=paste(methodname,".QQ.Plot.jpeg",sep = ""),
			width=600*5,height=600*5,res=72*4)
	par(mfrow=c(1,1),mar=c(3.1,3.1,0.7,1),oma=c(2.5,2.5,2.5,0),
			tcl=-0.5,mgp=c(2.8,1.3,0),lwd = 1.5)
	P.values <- as.matrix(pValue)
```

```
	N=nrow(P.values)
	P.values <- as.matrix((P.values)[order(P.values)])
	log.P.values <- as.matrix(rev(P.values))
	p_value_quantiles <- (1:N)/(N+1)
	log.Quantiles <- -log10(p_value_quantiles)
	N1=length(log.Quantiles)
	c95 <- rep(NA,N1)
	c05 <- rep(NA,N1)
	for(j in 1:N1){
		k=ceiling((10^-log.Quantiles[j])*N)
		if(k==0)k=1
		c95[j] <- qbeta(0.95,k,N-k+1)
		c05[j] <- qbeta(0.05,k,N-k+1)
	}
	plot(NULL, xlim = c(0,max(log.Quantiles)),
			ylim = c(0,max(c(log.P.values,-log10(c05)))),
			cex.axis=3.0, cex.lab=2.2, type="l",lty=1, lwd = 5,
			axes=TRUE, xlab="", ylab="",col="gray",yaxt ="n",xaxt ="n")
	index=length(c95):1
	polygon(c(log.Quantiles[index],log.Quantiles),c(-log10(c05)[index],-log10(c95)),
		col='gray',border=NA)
	abline(a = 0, b = 1, col = "black",lwd=2)
	color <- c("navy")
	points(log.Quantiles, log.P.values ,col=color,cex=1.1)
	x.lim <- max(log.Quantiles)
	y.lim <- max(c(log.P.values,-log10(c05)))
	axis(1,at=1:ceiling(x.lim),cex.axis=2.3,
			labels=c(1:ceiling(max(x.lim))),tick=TRUE,lwd.ticks=3)
	axis(2,at=seq(1,ceiling(y.lim),3),cex.axis=2.3,
			labels=seq(1,ceiling(y.lim),3),tick=TRUE,lwd.ticks=3)
	box()
	palette("default")
	mtext(expression(Observed~~-log[10](italic(p))),side=2,cex=1.9,outer=TRUE,line=-0.4)
	mtext(expression(Expected~~-log[10](italic(p))),side=1,cex=1.9,outer=TRUE)
	dev.off()
}

Manh_plot <- function(methodname,manh){
	jpeg(file=paste(methodname,".Manhattan.Plot.Genomewise.jpeg",sep = ""),
			width=900*5,height=500*5,res=72*4)
	par(mfrow=c(1,1),mar=c(3.1,3.1,0.7,1),oma=c(2.5,2.5,2.5,0),
			tcl=-0.5,mgp=c(2.8,1.3,0),lwd = 1.5)
	manh0 <- manh
	cutOff <- 0.05
	manh <- matrix(as.numeric(as.matrix(manh0)),nrow(manh0),ncol(manh0))
	manh <- manh[manh[,1]!=0,]
	numMarker <- nrow(manh)
	bonferroniCutOff <- -log10(cutOff/numMarker)
```

```
        y.lim <- ceiling(max(manh[,3]))
        chmtoanalyze <- unique(manh[,1])
        nchr <- length(chmtoanalyze)
        chrcolor <- c("forestgreen","firebrick2","royalblue2","orangered1",
                "gold2","violetred2","black")
        plotcolor <- rep(chrcolor,ceiling(nchr/5))
        mypch=20
        manh <- manh[order(manh[,2]),]
        manh <- manh[order(manh[,1]),]
        ticks=NULL
        lastbase=0
        for (i in chmtoanalyze){
                index=(manh[,1]==i)
                ticks <- c(ticks, lastbase+mean(manh[index,2]))
                manh[index,2]=manh[index,2]+lastbase
                lastbase=max(manh[index,2])
        }
        x <- as.numeric(manh[,2])
        y <- as.numeric(manh[,3])
        z <- as.numeric(manh[,1])
        size=1;ratio=10;base=1
        themax=ceiling(max(y))
        themin=floor(min(y))
        wd=((y-themin+base)/(themax-themin+base))*size*ratio
        s=size-wd/ratio/2
        plot(y~x,xlab="",ylab="" ,ylim=c(0,y.lim),cex.axis=2.1, cex.lab=2.2,col=plotcolor[z],
                        axes=FALSE,type = "p",pch=mypch,lwd=wd,cex=s+.5,main = "",cex.main=2)
        abline(h=bonferroniCutOff,col="forestgreen")
        axis(1, at=ticks,cex.axis=2.3,labels=chmtoanalyze,tick=TRUE,lwd.ticks = 3)
        axis(2, at=seq(1,floor(y.lim),3),cex.axis=2.3,
                        labels=seq(1,floor(y.lim),3),tick=TRUE,lwd.ticks = 3)
        box()
        palette("default")
        mtext(expression(Observed~~-log[10](italic(p))),side=2,cex=1.9,outer=TRUE,line=-0.4)
        mtext(expression(Chromosome),side=1,cex=1.9,outer=TRUE)
        dev.off()
}

###################### Main programm #####################
###  Load R packages
library(RcppArmadillo)
library(snow)
library(MASS)

###  Data input
y0 <- read.csv("Phenotype.csv", header=TRUE)
g0 <- read.table("genotype.txt", header=FALSE, sep="\t")
freq <- read.table("freq.txt", header=FALSE, sep="\t")
```

```
gname_chr_pos_info <- read.table("gname_chr_pos_info.txt", header=TRUE)
N_gene <- nrow(gname_chr_pos_info)
bonfCut <- -log10(0.05/N_gene)

################################################################################
#                          GWAS Circulation for Traits                         #
################################################################################
for (T in 2:ncol(y0)){
        y<-y0[T]
        y<-as.matrix(y)
        name <-colnames(y0[T])
        nobs <- nrow(g0)
        nmar <- ncol(g0)

        ##############################################################
        #                   Spectral decomposition                   #
        ##############################################################
        A <- scale(g0)
        B <- t(A)
        cl <- makeSOCKcluster(rep("localhost",20))
        G <- parMM(cl, A, B)
        stopCluster(cl)
        G <- G/nmar
        sg <- eigen(G)$values
        ug <- eigen(G)$vectors
        step <- 1
        hh <- seq(0,0.999,0.001*step)
        hh <- round(hh,3)
        sg <- matrix(rep(sg,length(hh)),nobs)
        V <- t(t(sg)*hh/(1-hh)) + 1
        logV0 <- apply(log(V),2,sum)
        V050 <- 1/sqrt(V)
        gnewb0 <- t(ug)%*%as.matrix(rep(1,nobs))
        ynew <- t(ug)%*%y

        ##############################################################
        #             GBLUP(by spectral transformation)              #
        ##############################################################
        fva0 <- GBLUP(ynew,as.matrix(gnewb0))
        hh0 <- fva0$h2
        ve0 <- fva0$ve

        ##############################################################
        #            EPC method based on Single-RunKing              #
        ##############################################################
        hpos <- which(hh==hh0)
        ynew1 <- V050*ynew[,1]
        ###  Analysis of genes
```

```
	k1 <- 1
	dp <- matrix(nrow=length(freq),ncol=5)
	for (i in 1:length(freq)){
		k2<-k1+freq[i]-1
		block<-g0[,k1:k2]
		eig <- eigen(cov(block))
		pc <- block%*%eig$vectors
		eigenval <- eig$values
		for(j in 1:length(eigenval)){
			cumulvar <- sum(eigenval[1:j])/sum(eigenval)
			if(cumulvar>0.80)break
		}
		x1 <- pc[,1:j]
		xnew <- t(ug)%*%x1
		fva <- fast_lmm_block(ynew1,cbind(gnewb0,xnew),1)
		dp[i,] <- c(i,fva)
		k1 <- k1+freq[i]
	}

	### GWAS result output
	colnames(dp) <- c("geneNo","chiqnew","Pvalue","Loglike","Fvalue")
	write.table(dp, paste("GWASresult_80pc_", name, ".txt", sep=""), row.names=FALSE,
col.names=TRUE)

	### QQ and Manhattan plot and significant genes
	manh_gene <- data.frame(gname_chr_pos_info, dp[,c(2,3)])
	manh_gene[,5] <- -log10(manh_gene[,5])
	if(length(which(is.infinite(manh_gene[,5])))!=0){
		print((which(is.infinite(manh_gene[,5]))))
		QQ_plot(paste("p_80pc_",name,sep=""), manh_gene[ ,5][-
which(is.infinite(manh_gene[,5]))])
		Manh_plot(paste("p_80pc_",name,sep=""), manh_gene[-
which(is.infinite(manh_gene[,5])),c(2,3,5)])
	}else{
		QQ_plot(paste("p_80pc_",name,sep=""), manh_gene[ ,5])
		Manh_plot(paste("p_80pc_",name,sep=""), manh_gene[ ,c(2,3,5)])
	}
	sig <- which(manh_gene[ ,5] > bonfCut)
	sig_gene_80pc <- manh_gene[sig, ]
	write.table(sig_gene_80pc, paste("sig_gene_80pc_",name,".txt",sep=""), col.names=TRUE,
row.names=FALSE)

}###T

########################## End ##############################
```
