# Supplementary figures and images for: Gene-Based Genome-Wide Association Study Identified Genes for Agronomic Traits in Maize
Source: Biology (Basel). 2022 Nov 11;11(11):1649. doi: 10.3390/biology11111649 (PMC9687540; doi:10.3390/biology11111649)

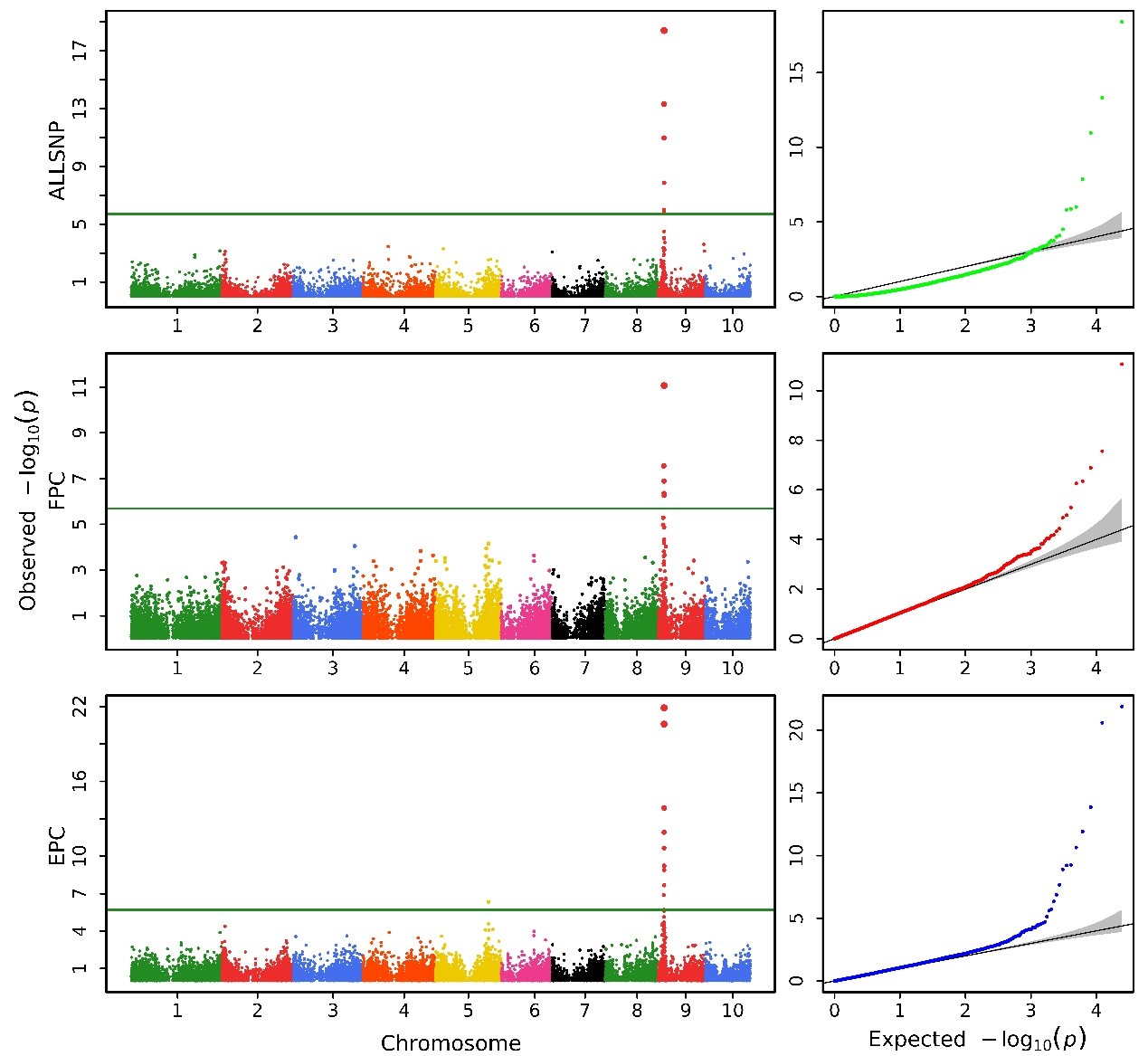

Supplement: Supplementary file 1 [file biology-11-01649-s001.zip › Supplementary Figures/Figure S1.jpg]

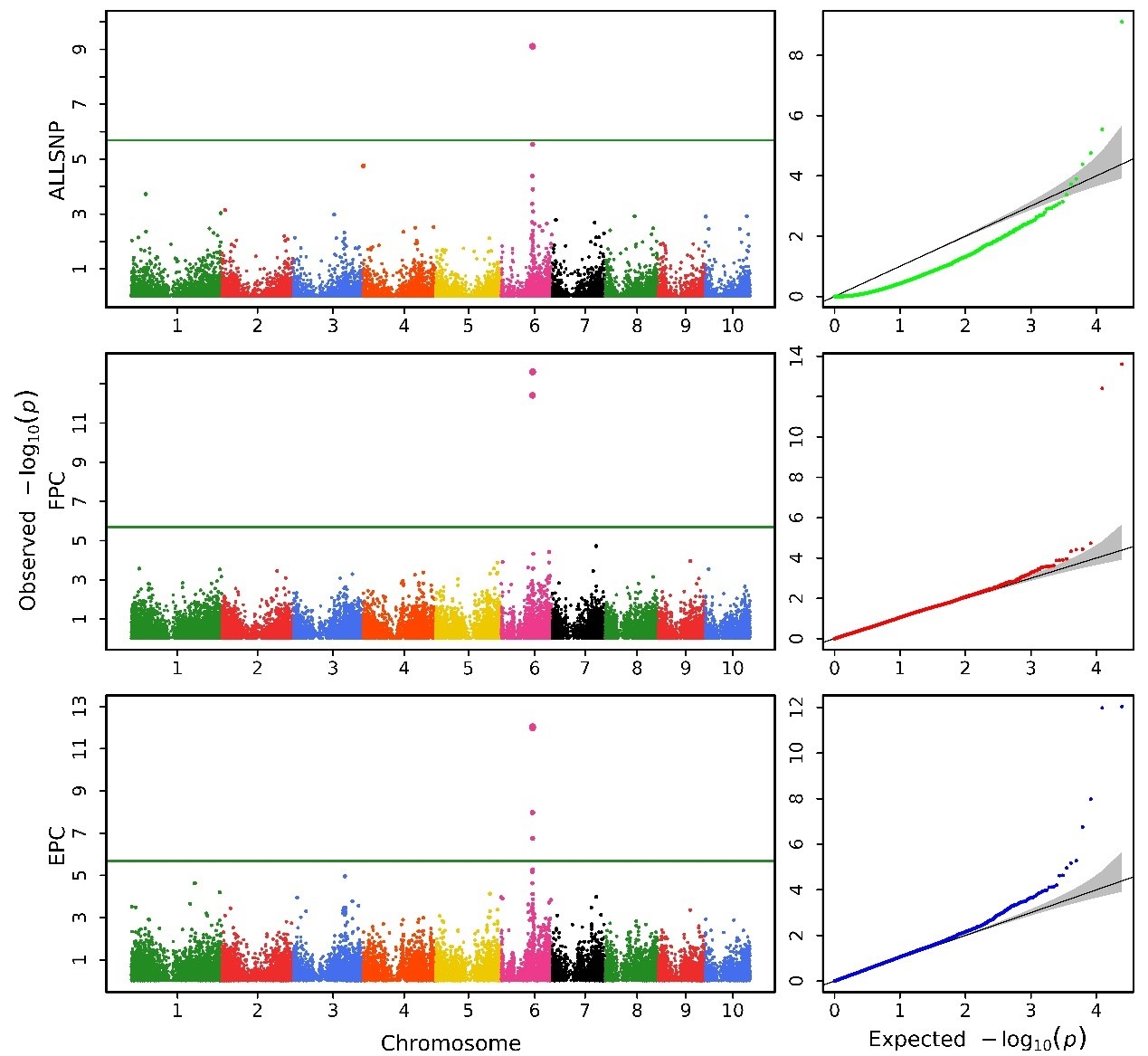

Supplement: Supplementary file 1 [file biology-11-01649-s001.zip › Supplementary Figures/Figure S10.jpg]

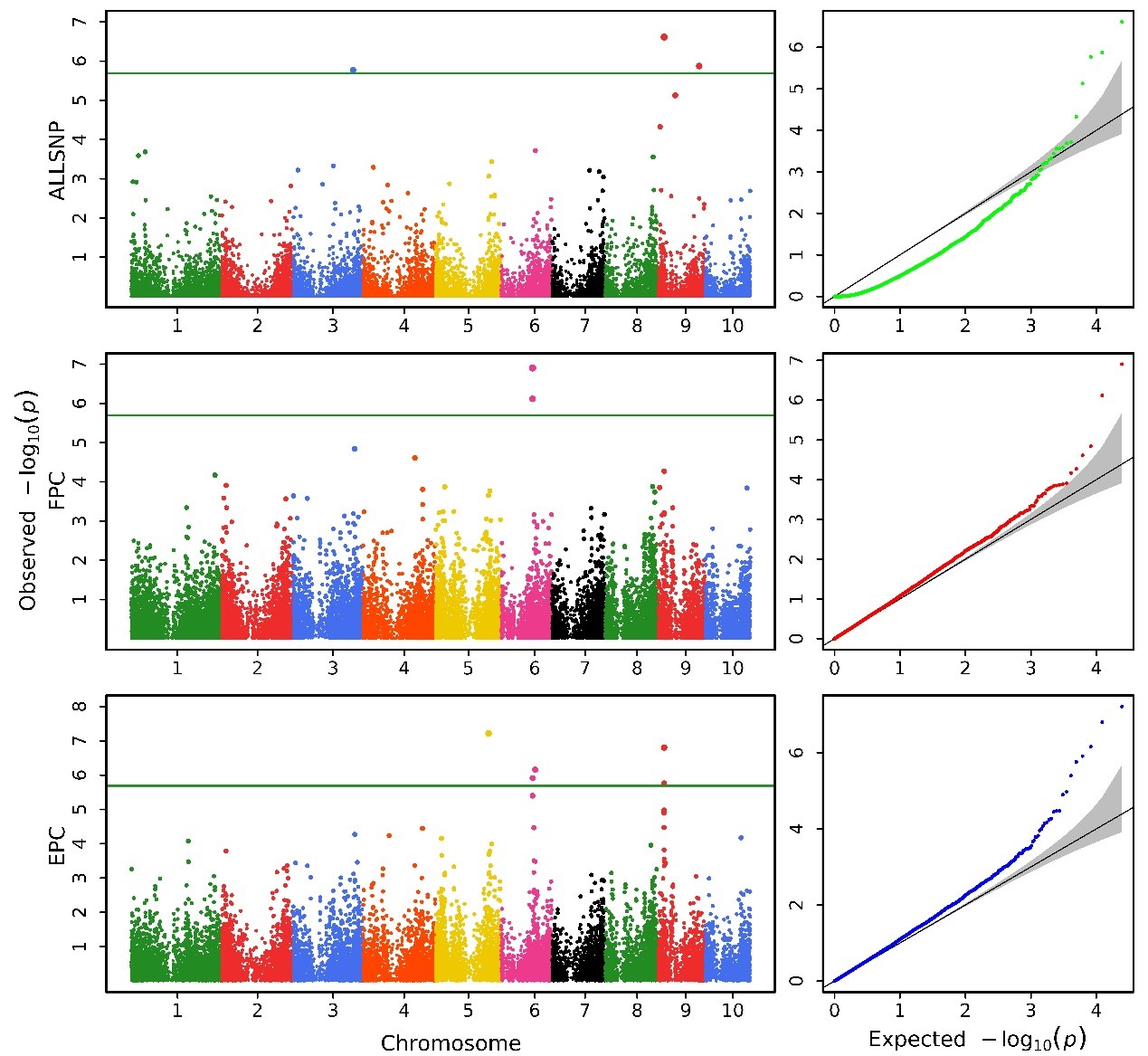

Supplement: Supplementary file 1 [file biology-11-01649-s001.zip › Supplementary Figures/Figure S11.jpg]

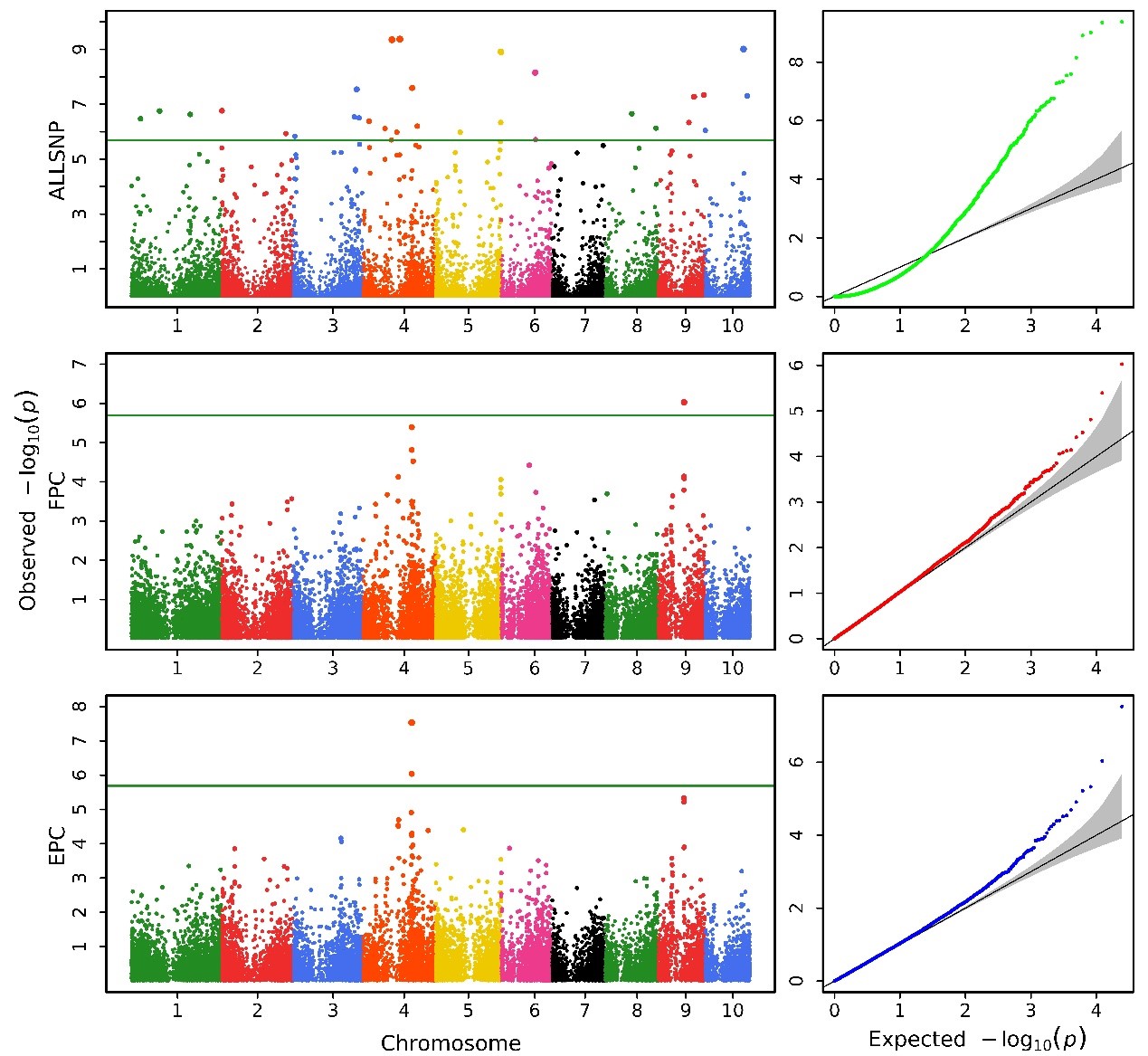

Supplement: Supplementary file 1 [file biology-11-01649-s001.zip › Supplementary Figures/Figure S12.jpg]

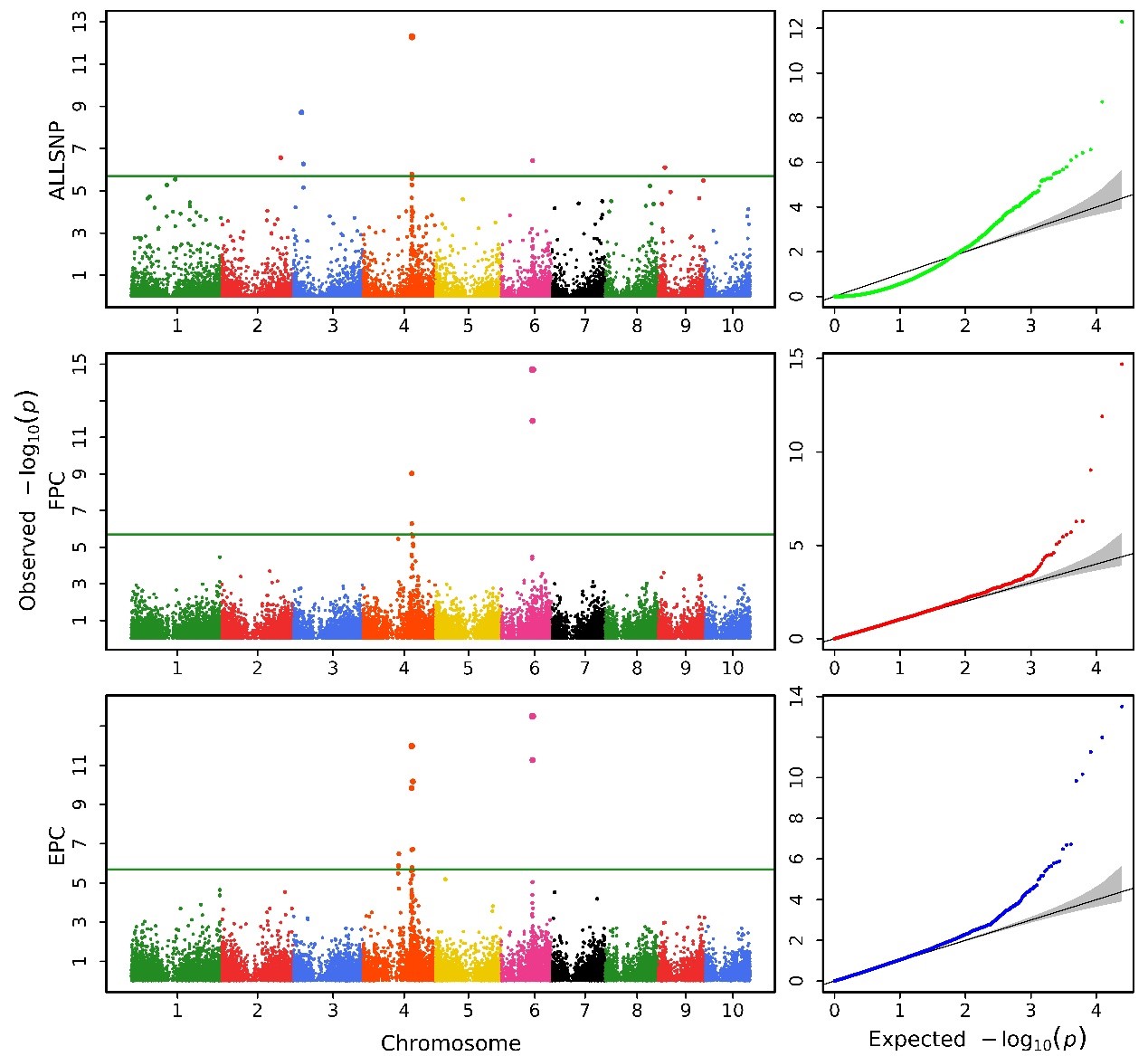

Supplement: Supplementary file 1 [file biology-11-01649-s001.zip › Supplementary Figures/Figure S13.jpg]

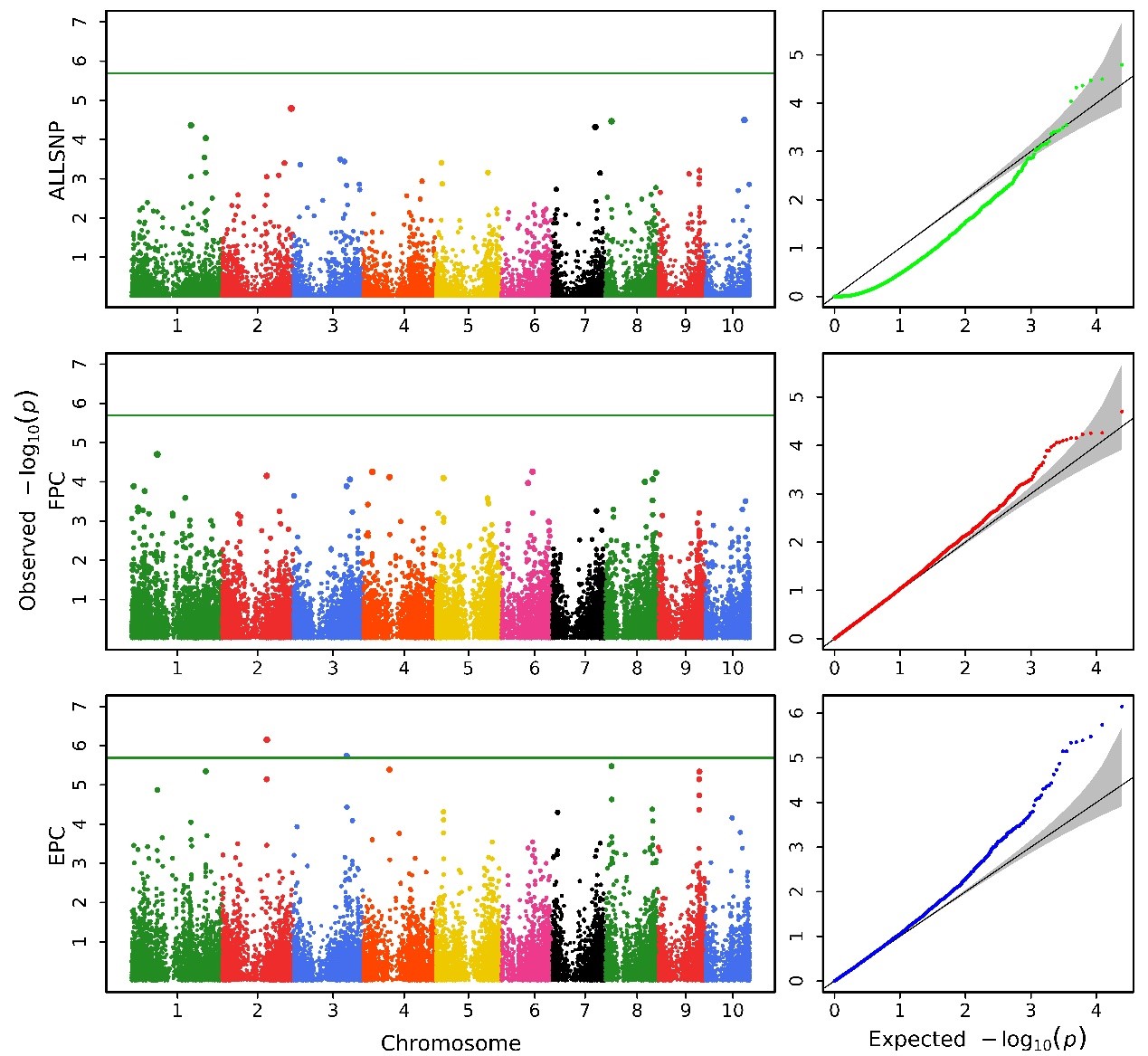

Supplement: Supplementary file 1 [file biology-11-01649-s001.zip › Supplementary Figures/Figure S14.jpg]

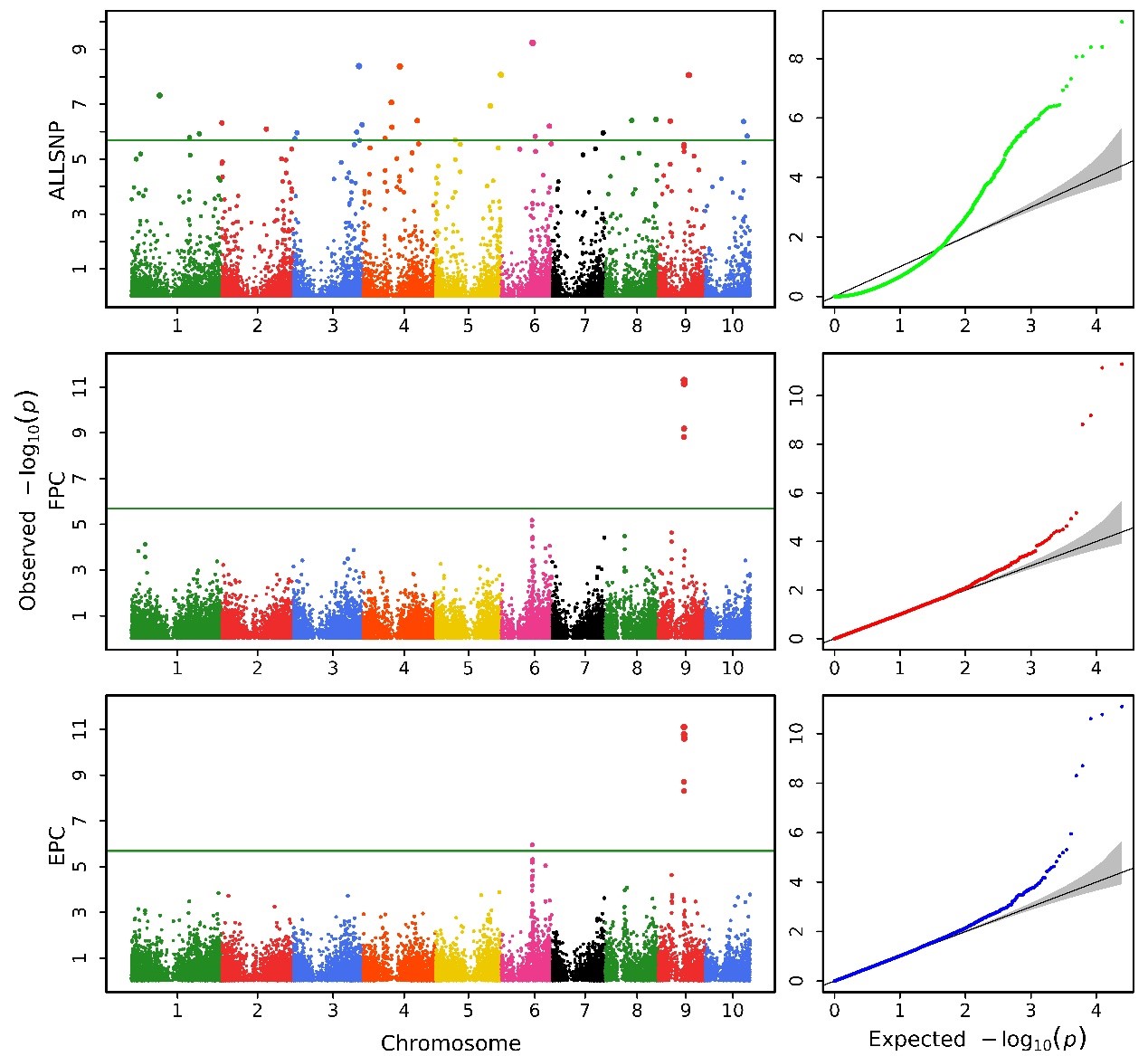

Supplement: Supplementary file 1 [file biology-11-01649-s001.zip › Supplementary Figures/Figure S15.jpg]

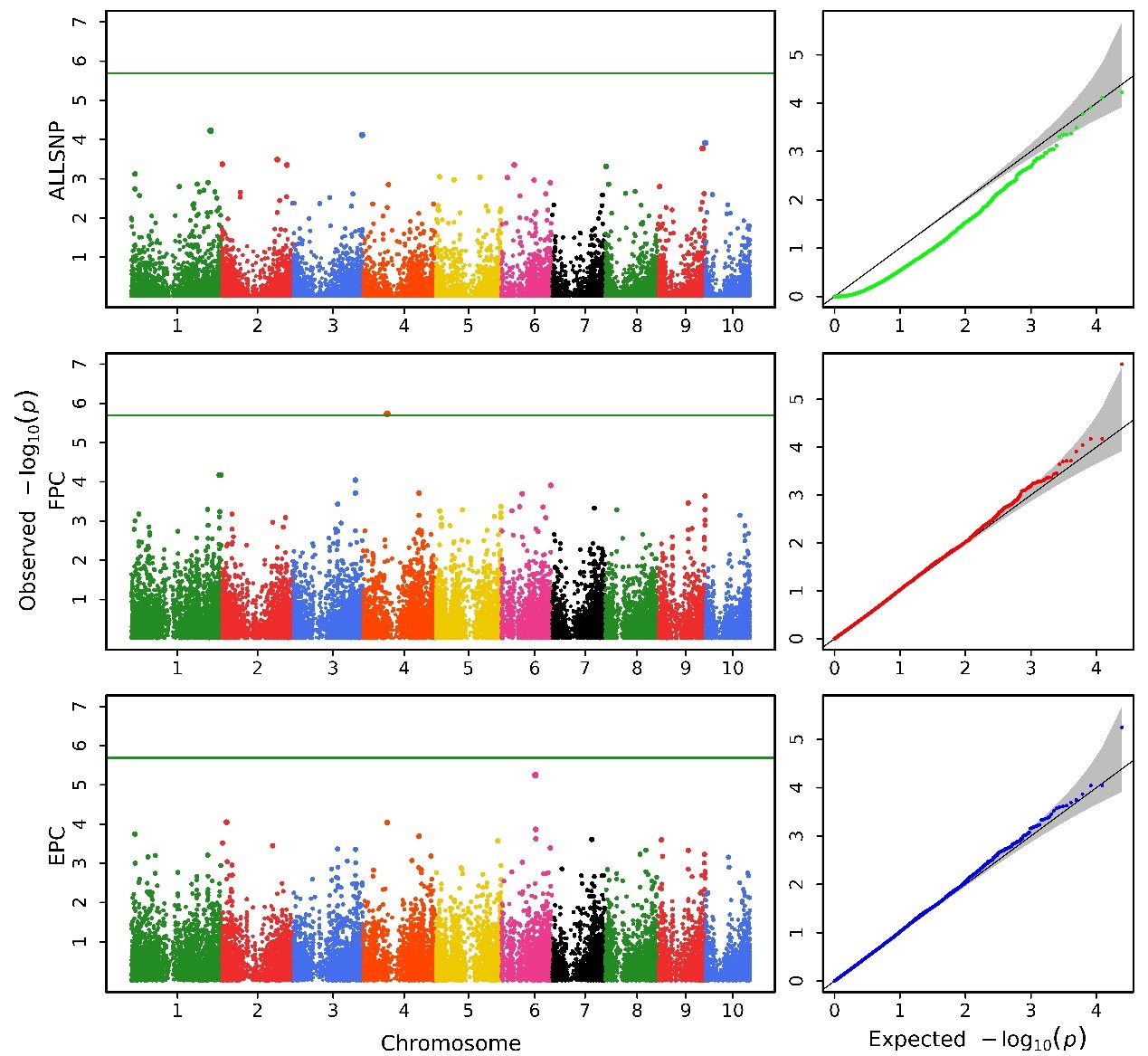

Supplement: Supplementary file 1 [file biology-11-01649-s001.zip › Supplementary Figures/Figure S16.jpg]

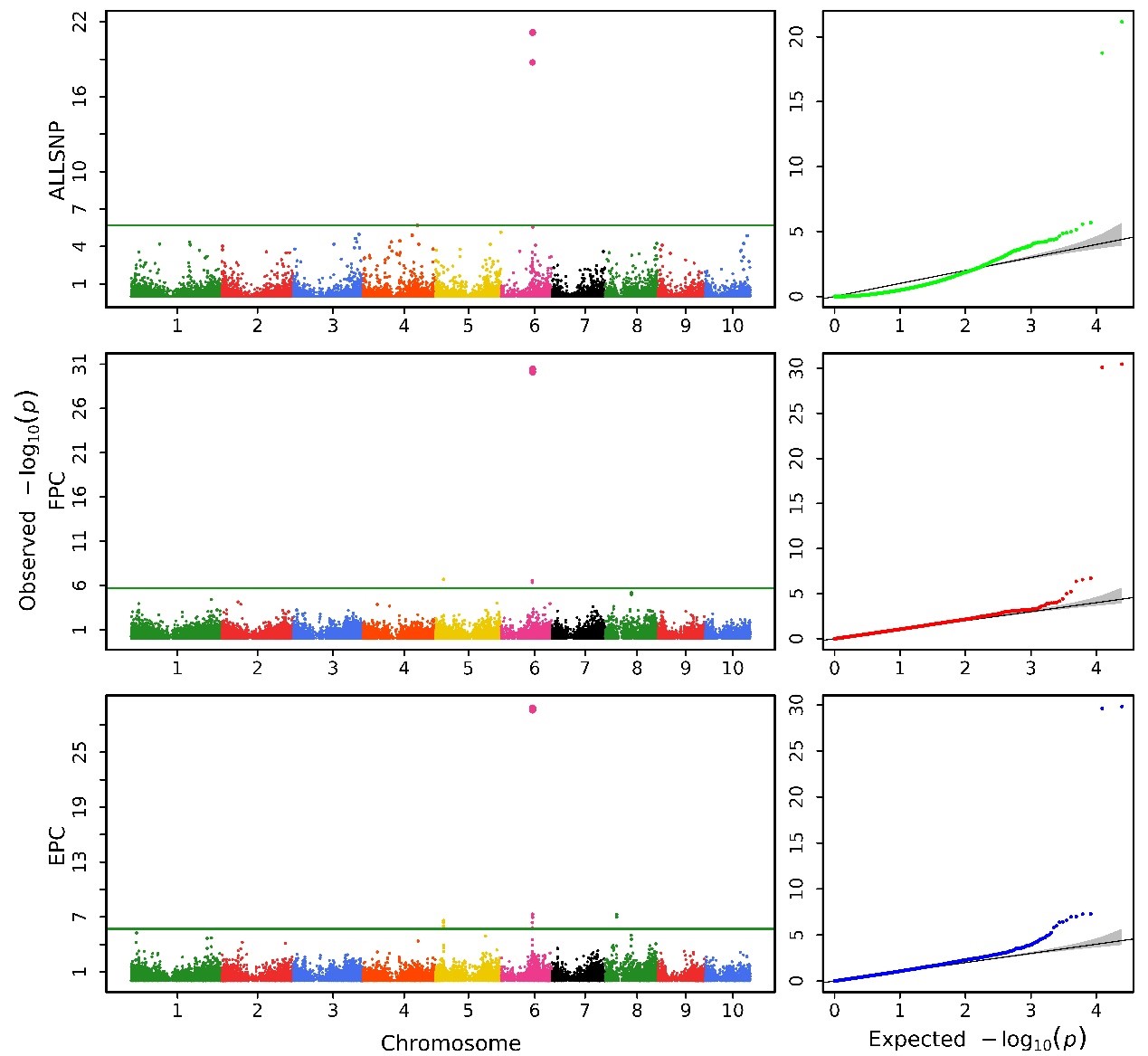

Supplement: Supplementary file 1 [file biology-11-01649-s001.zip › Supplementary Figures/Figure S17.jpg]

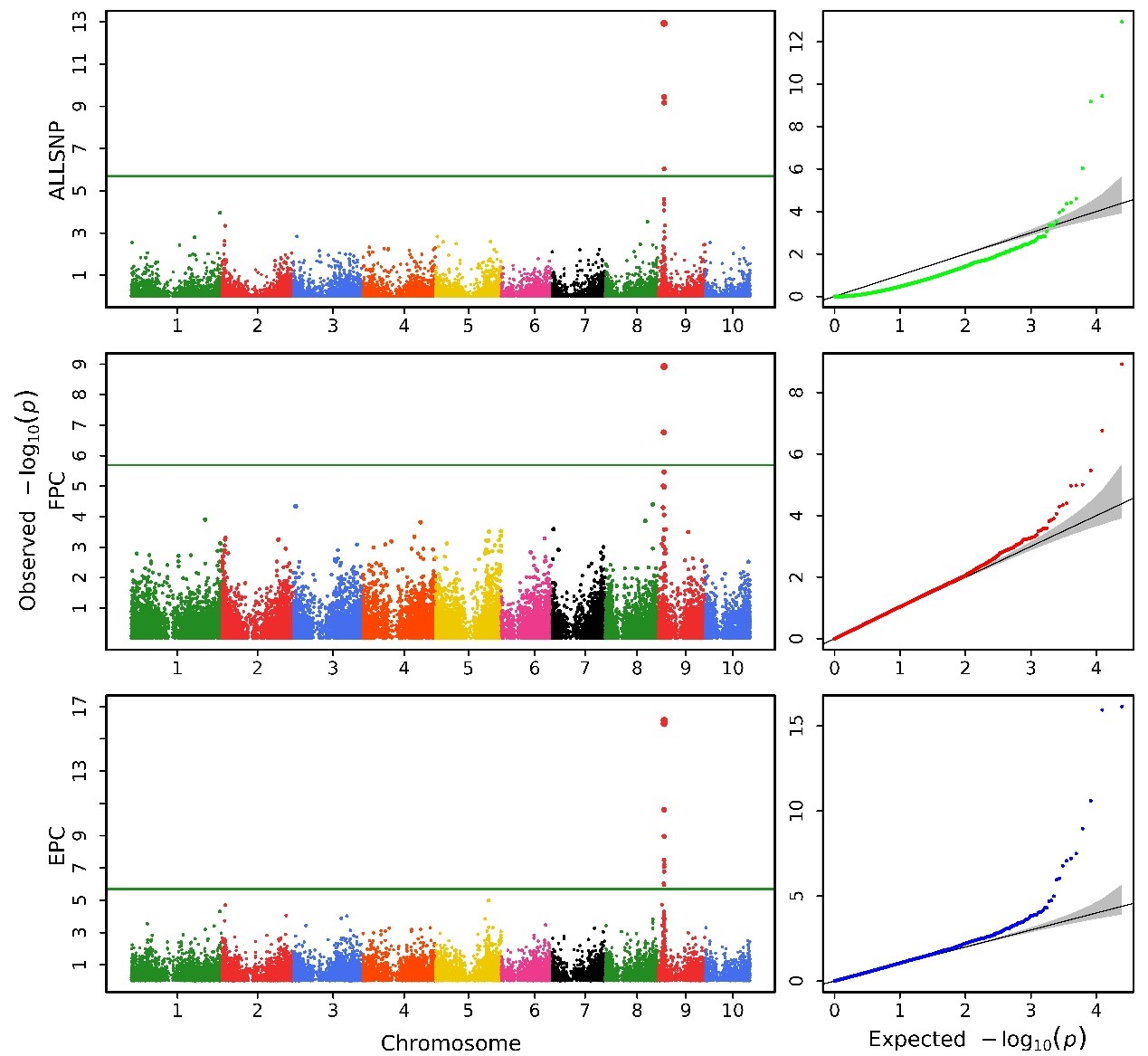

Supplement: Supplementary file 1 [file biology-11-01649-s001.zip › Supplementary Figures/Figure S18.jpg]

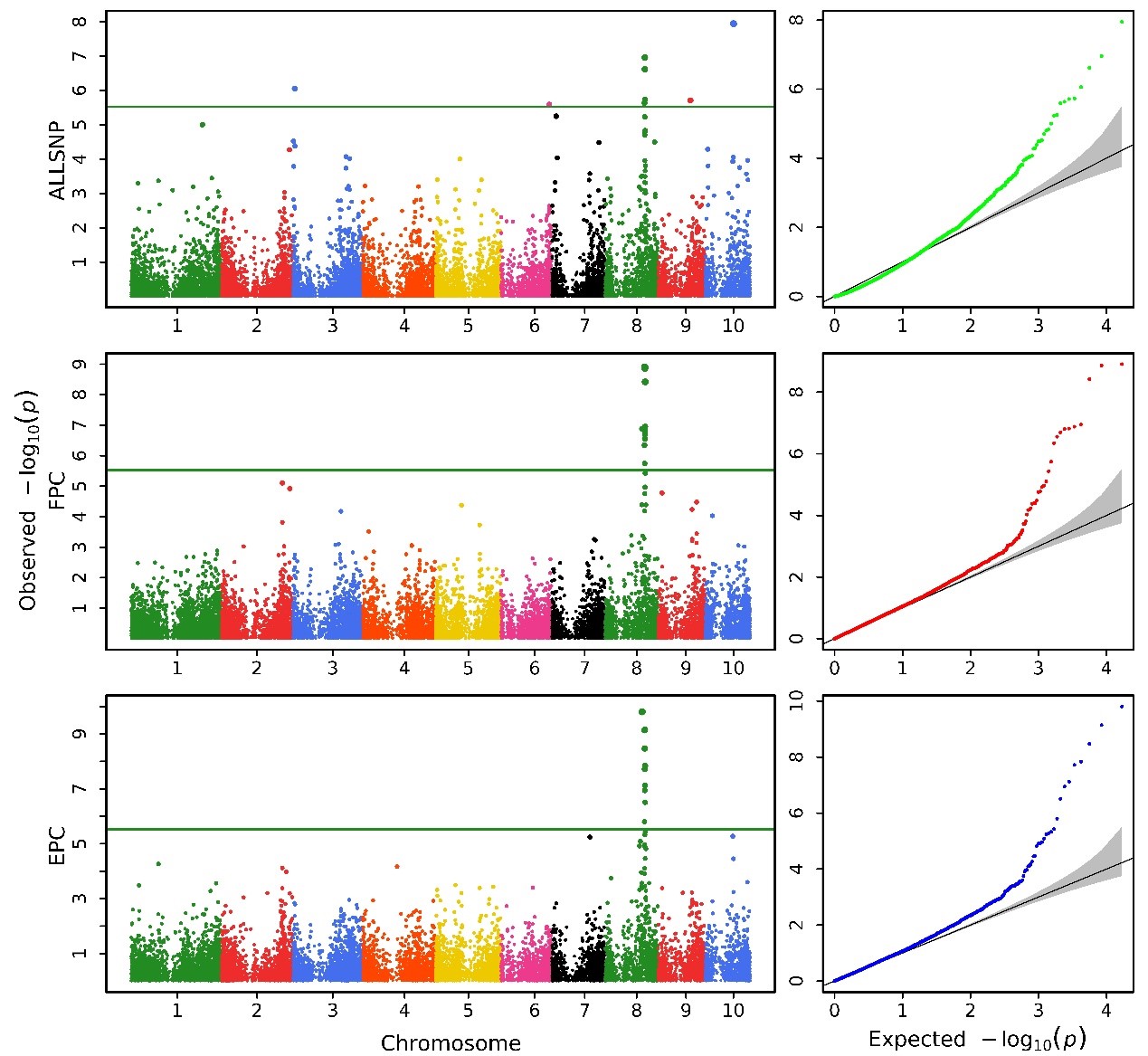

Supplement: Supplementary file 1 [file biology-11-01649-s001.zip › Supplementary Figures/Figure S19.jpg]

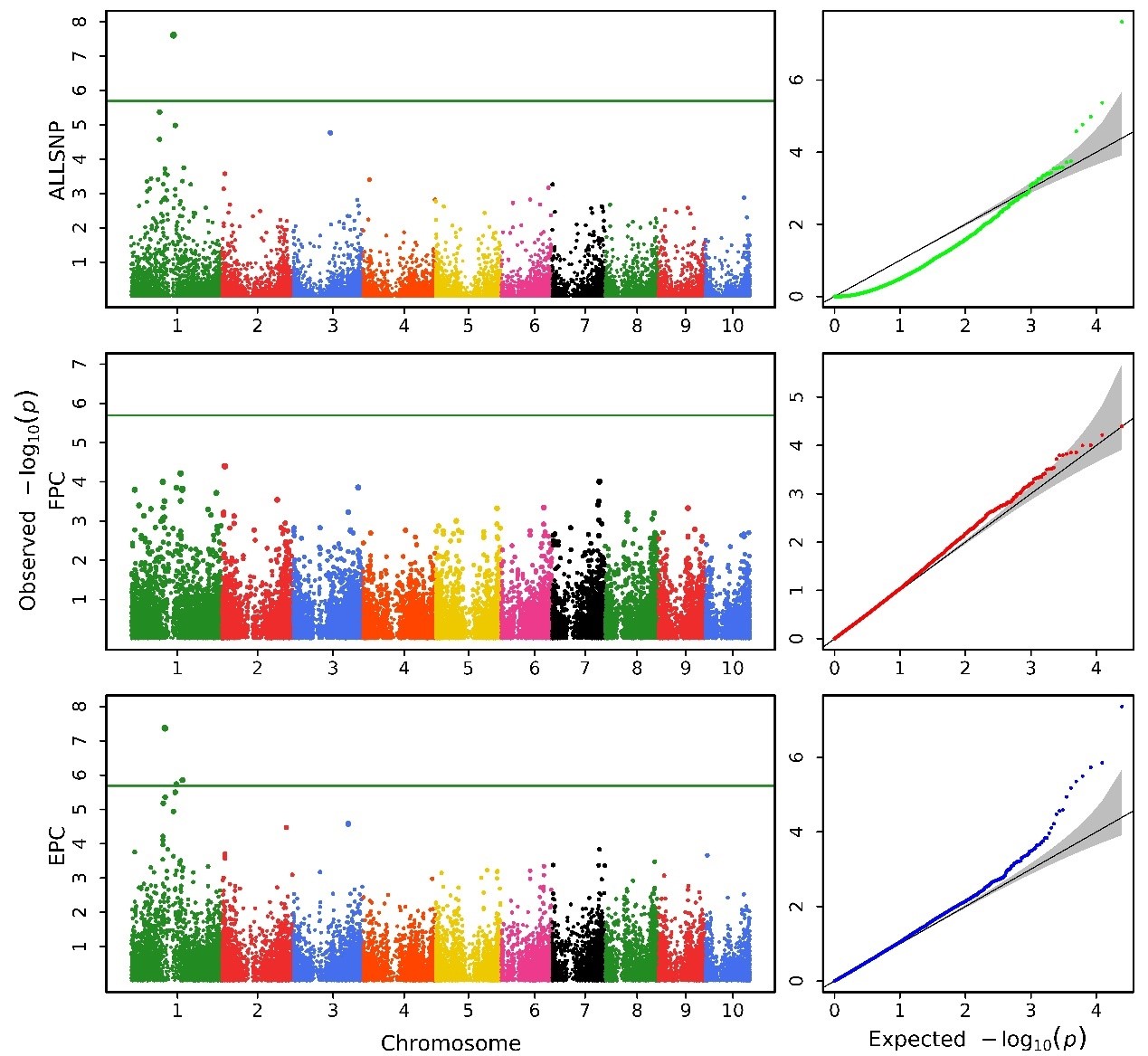

Supplement: Supplementary file 1 [file biology-11-01649-s001.zip › Supplementary Figures/Figure S2.jpg]

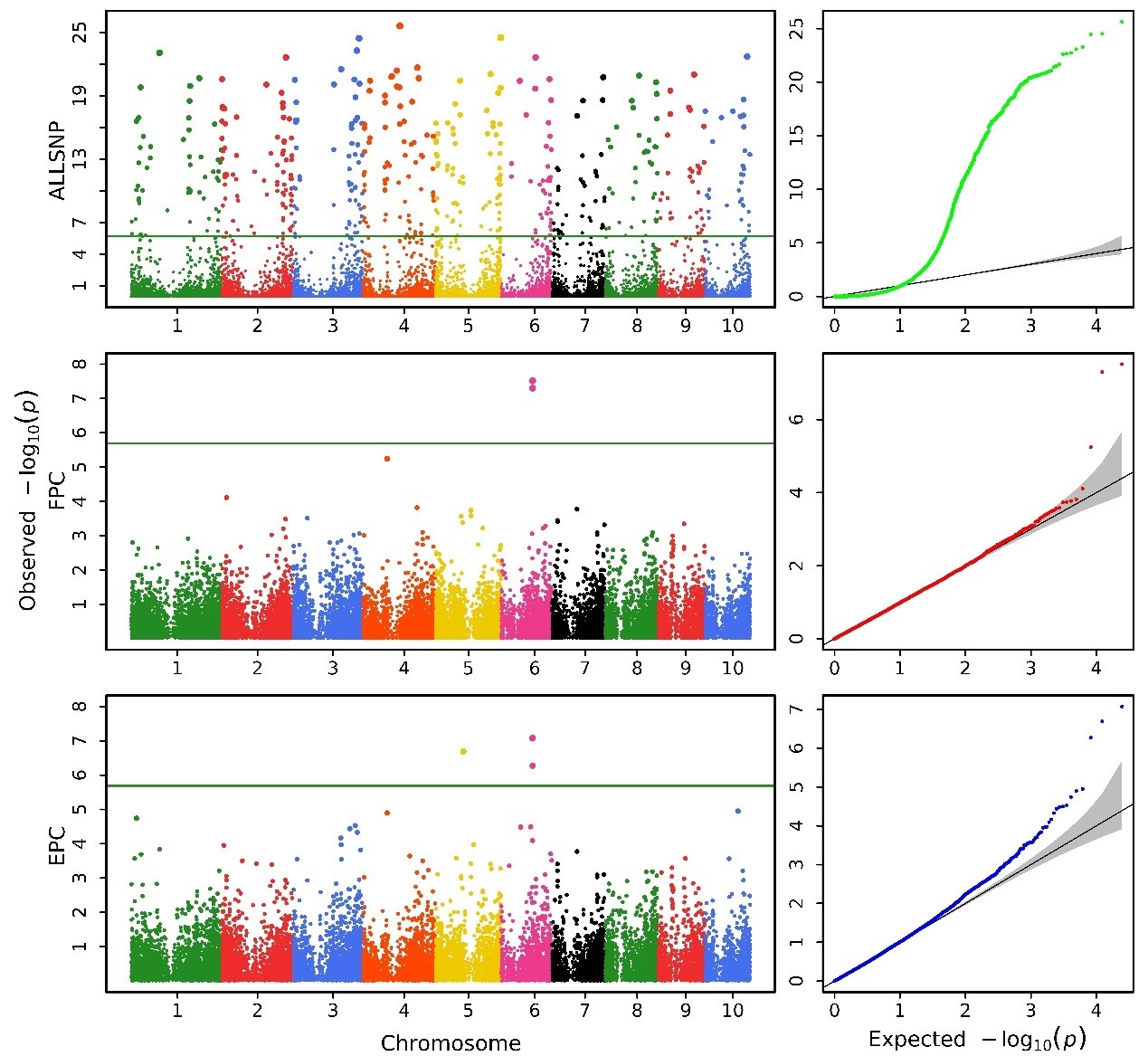

Supplement: Supplementary file 1 [file biology-11-01649-s001.zip › Supplementary Figures/Figure S3.jpg]

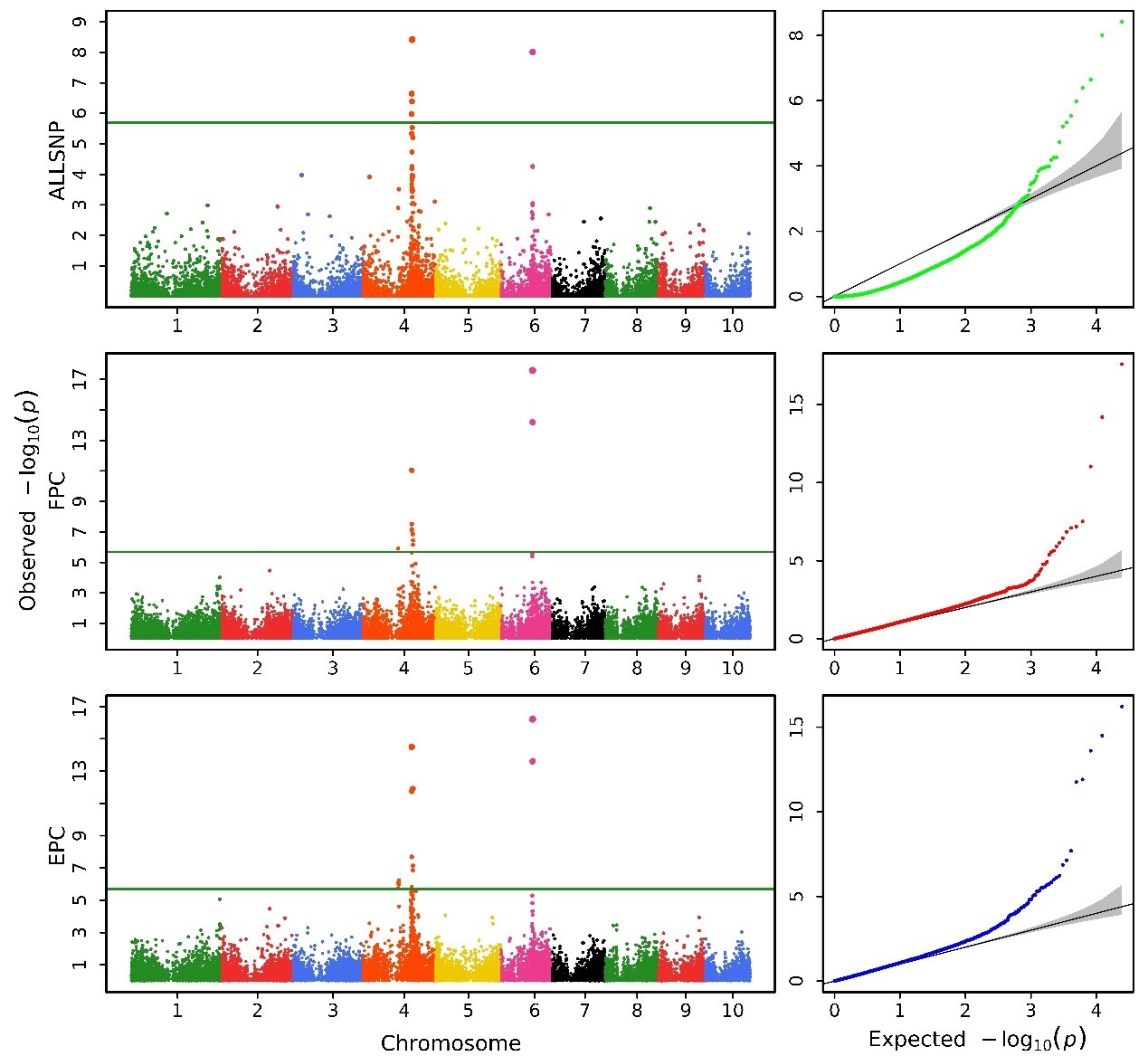

Supplement: Supplementary file 1 [file biology-11-01649-s001.zip › Supplementary Figures/Figure S4.jpg]

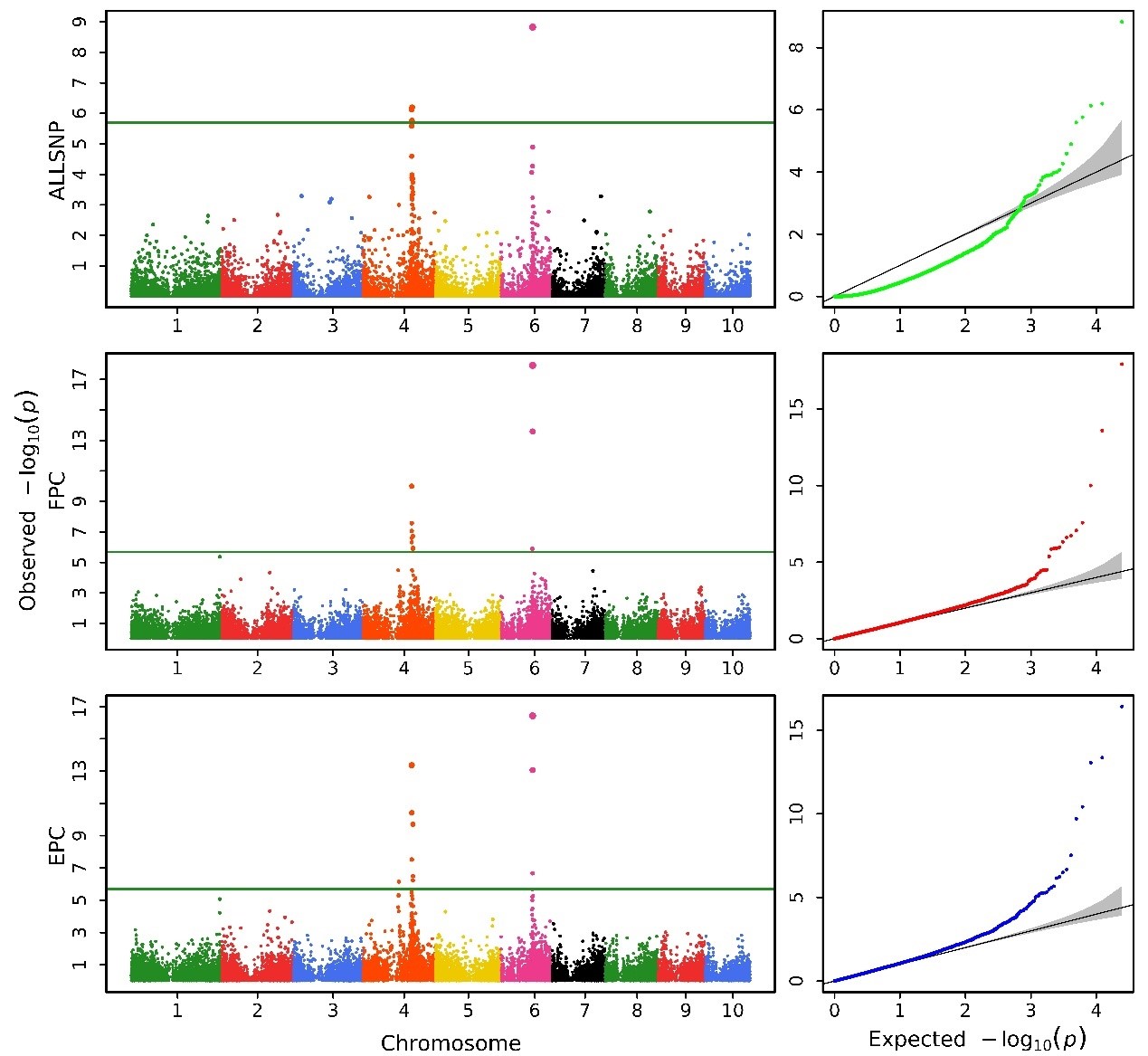

Supplement: Supplementary file 1 [file biology-11-01649-s001.zip › Supplementary Figures/Figure S5.jpg]

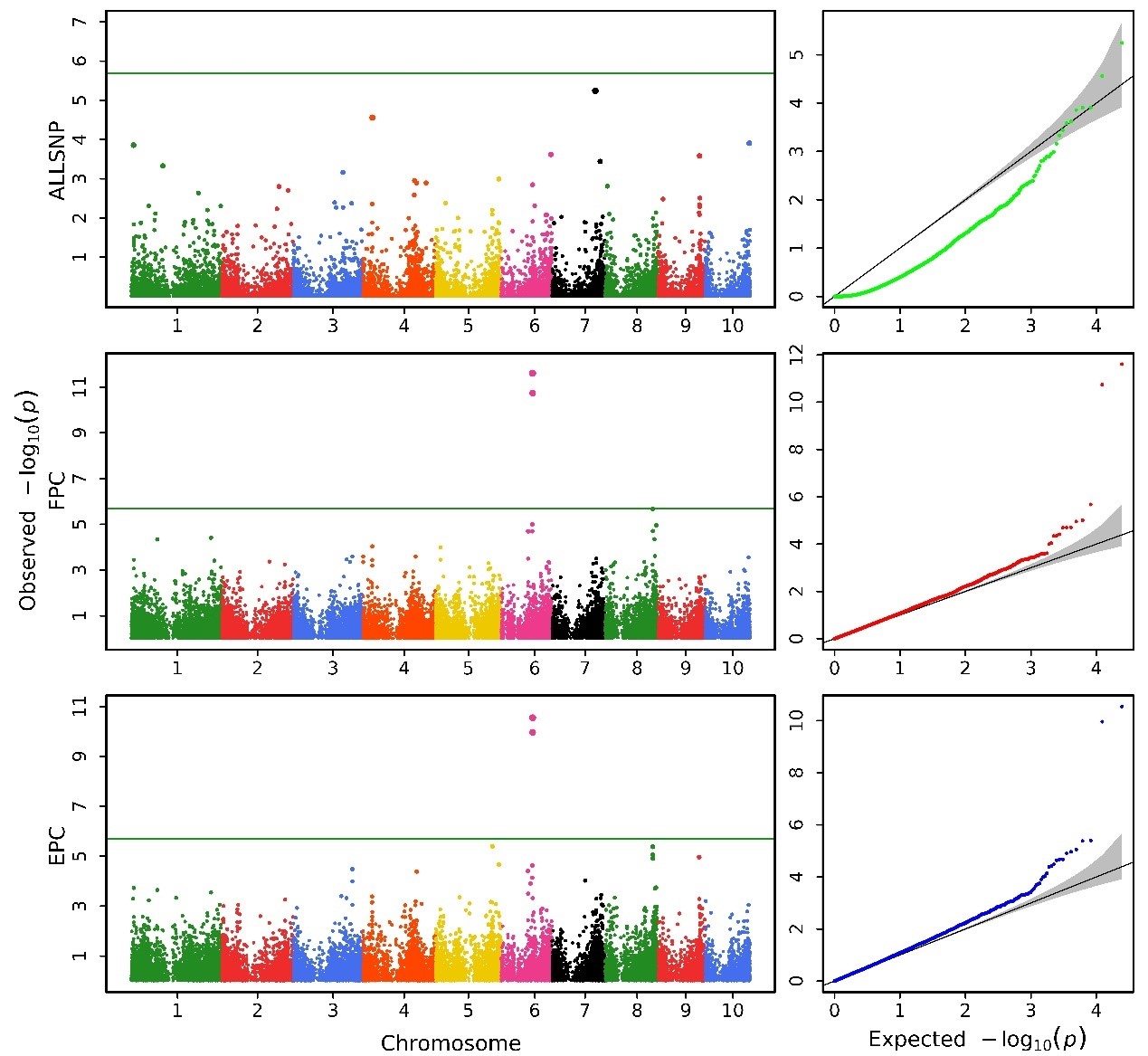

Supplement: Supplementary file 1 [file biology-11-01649-s001.zip › Supplementary Figures/Figure S6.jpg]

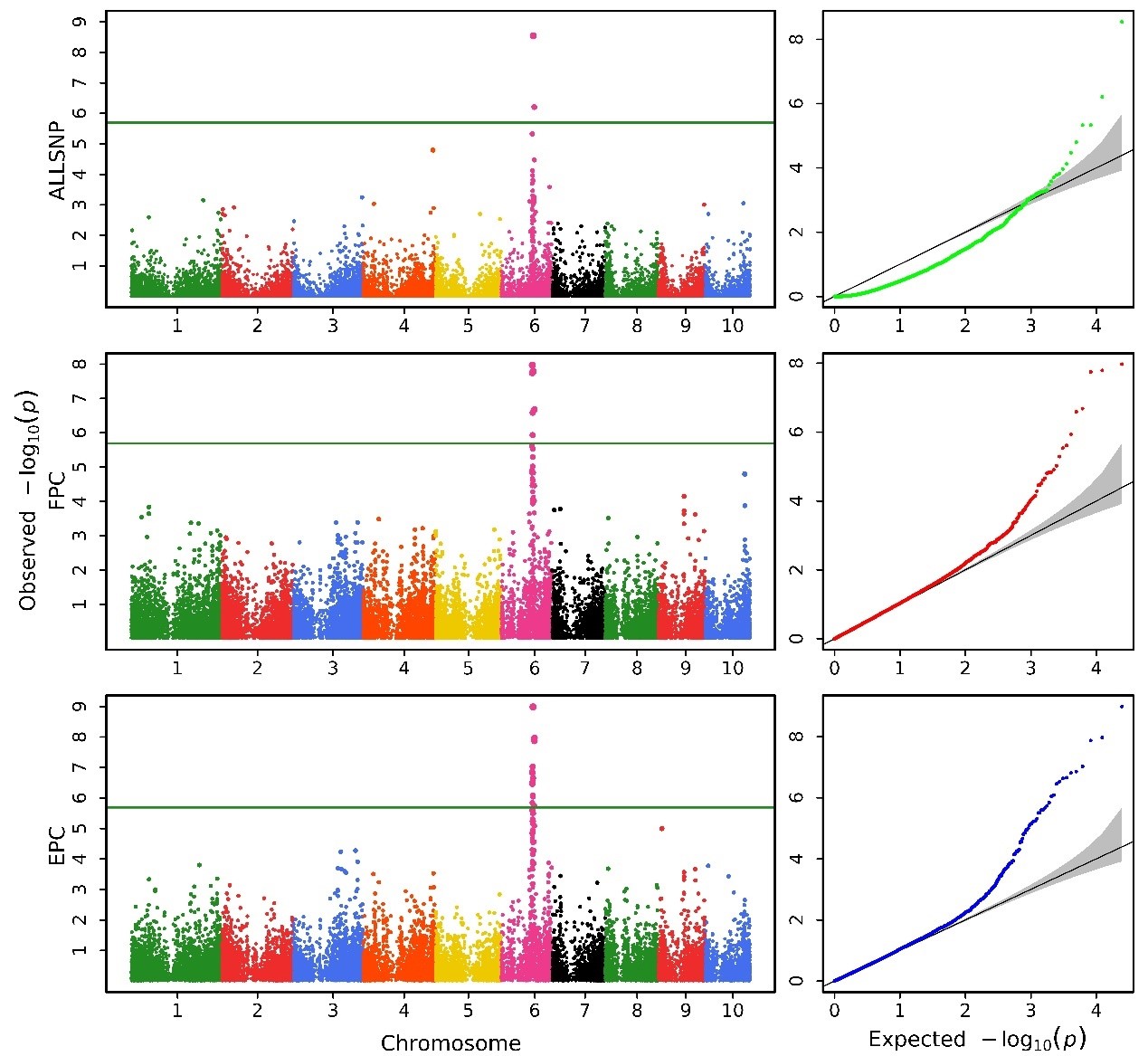

Supplement: Supplementary file 1 [file biology-11-01649-s001.zip › Supplementary Figures/Figure S7.jpg]

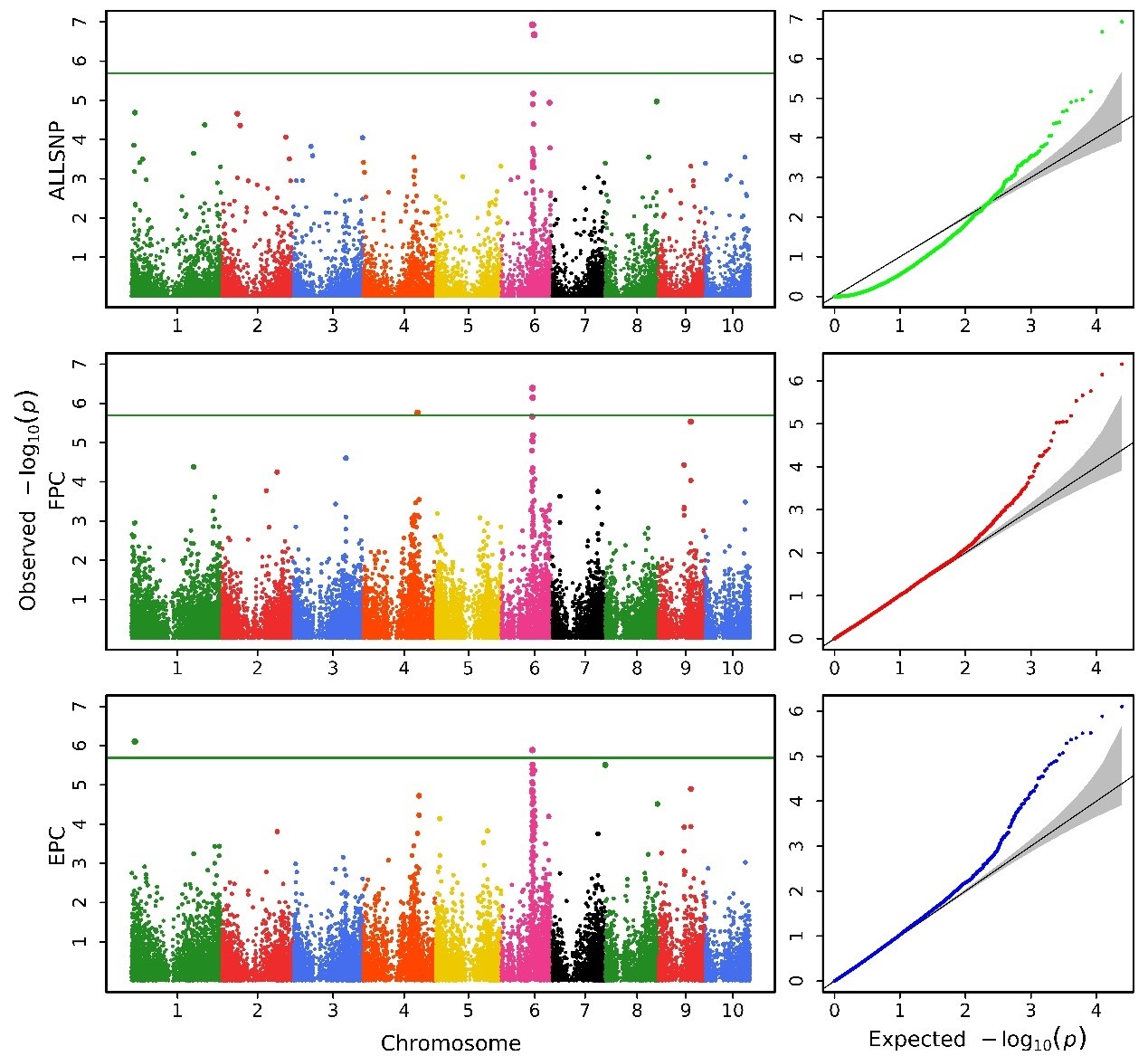

Supplement: Supplementary file 1 [file biology-11-01649-s001.zip › Supplementary Figures/Figure S8.jpg]

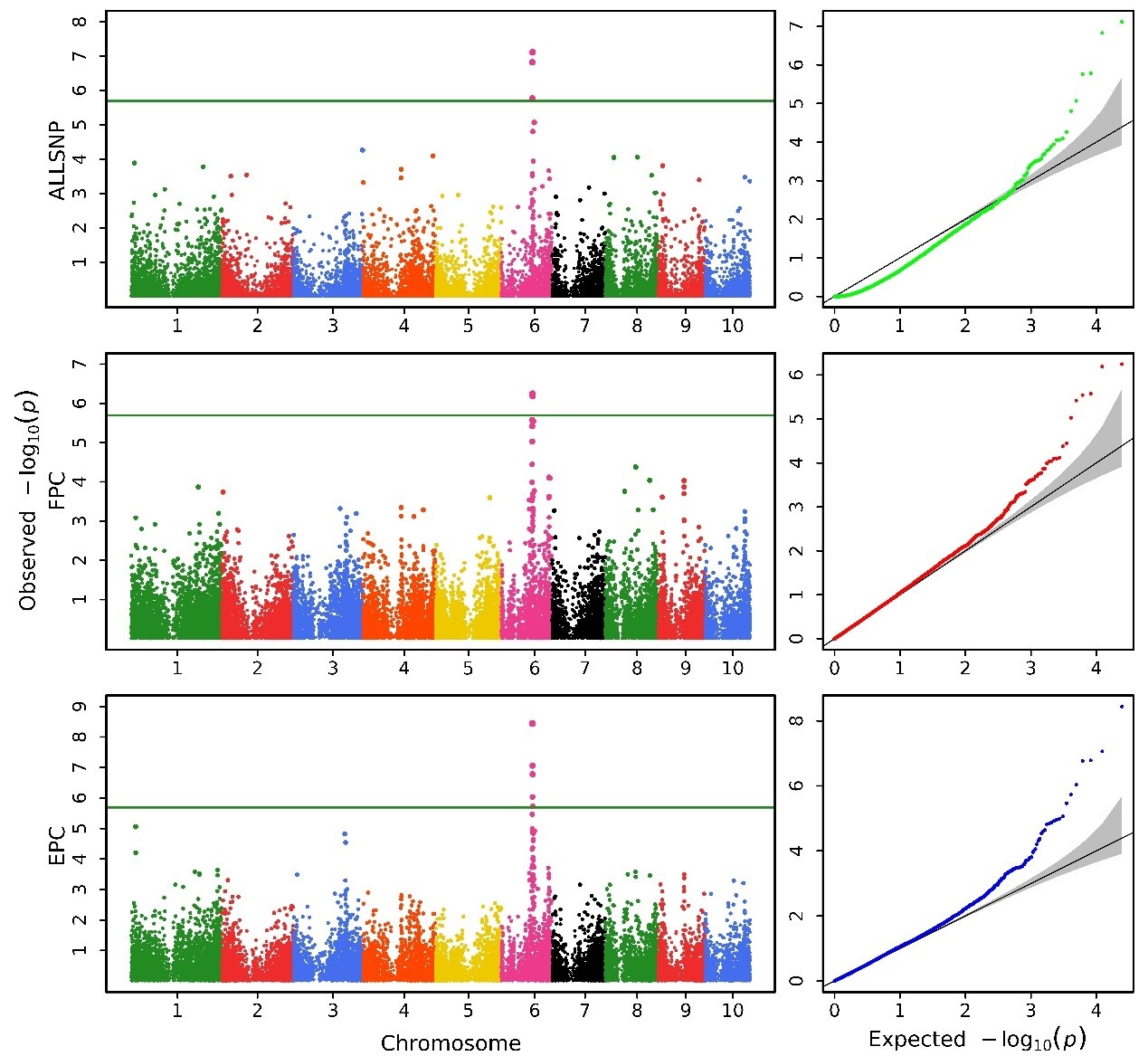

Supplement: Supplementary file 1 [file biology-11-01649-s001.zip › Supplementary Figures/Figure S9.jpg]
